# Supplementary material for: Sequence-sensitive elastic network captures dynamical features necessary for miR-125a maturation
Source: PLoS Comput Biol. 2022 Dec 14;18(12):e1010777. doi: 10.1371/journal.pcbi.1010777 (PMC9797095; doi:10.1371/journal.pcbi.1010777)
Supplement: S1 Text — Fig A. Correlation between miR-125a maturation efficiency and Dynamical Signature distance to WT for both ENCoM and MD simulations. Fig B. Cut-ANM and PD-ANM parameter sweep for the B-factors benchmark. Fig C. Performance of multiple linear regression on an 80–20 train-test split. Fig D. Size distributions of the sequence clusters from the 3 benchmarks. Fig E. Number of nontrivial normal modes at 5% for the overlap and NMR benchmarks. Fig F. Simulation of predictive R-squared from combinations of MC-Fold enthalpy with random noise. Fig G. Computational cost of the ENMs on the Thermus thermophilus 30S ribosomal subunit and on miR-125a. Table H. Atom type assignation of the four standard nucleotides. Table I. PDB codes of the structures used for the three benchmarks. Table J. Performance on individual structures for the B-factors correlation benchmark. Table K. Performance on individual pairs of conformations for the X-ray conformational change benchmark. Table L. Performance on individual NMR ensembles for the ensemble variance benchmark. Table M. MD trajectories of miR-125a variants. Table N. ENCoM interaction strength for different base pairs. (PDF) [file pcbi.1010777.s001.pdf]

Supplementary information for: Sequence-sensitive elastic network captures dynamical features necessary for miR-125a maturation

Olivier Mailhot[1−4] Vincent Frappier[5] François Major[2,3,*]
Rafael J. Najmanovich[4,*]

[1]Department of Biochemistry and Molecular Medicine, Université de Montréal, Montreal, Canada
[2]Department of Computer Science and Operations Research, Université de Montréal, Montreal, Canada
[3]Institute for Research in Immunology and Cancer, Université de Montréal, Montreal, Canada
[4]Department of Pharmacology and Physiology, Université de Montréal, Montreal, Canada
[5]Generate Biomedicines, Cambridge, Massachusetts
[*]To whom correspondence should be addressed.
December 6, 2022

Contact: rafael.najmanovich@umontreal.ca, francois.major@umontreal.ca

1 MOLECULAR DYNAMICS SIMULATIONS

We performed molecular dynamics simulations for the 16 possible miR-125a variants at base pair 22, the position of the G22U population SNP leading to poor prognisis in breast cancer [1]. We used the Chen-Garcia forcefield [2] with the GROMACS software [3] to accumulate more than 100ns for three replicate trajectories per variant, for a total of 5.75μs simulation data. Table M gives the simulation time of every individual trajectory. The starting structures were generated from MC-Sym model 22 because previous analyses had shown this model to lead to statistically significant performance in predicting maturation efficiency from the Dynamical Signature distance. This analysis, as well as the MD simulations, were performed before we developed the novel approach of using the full Dynamical Signature inside LASSO regression.

Starting from the *in silico* structures mutated using ModeRNA [4], we used a standard cubic box with edges 1nm away from the miR-125a structure, solvated the RNA using TIP4P water following personal communication with Alan Chen. 100mM NaCL was added to the system, in addition to the necessary amount of cations to make the system neutral. Energy minimization was performed until the biggest force on any atom was under 1 kJ/mol, followed by NVT and NPT equilibration for 100ps each, at 310K (human body temperature). The simulation was then carried until at least 100 ns of simulation time had been acquired, also at 310K, with a 2fs time-step. The hardware used was the Intel Gold 6148 Skylake 20-core CPU @ 2.4GHz, as part of Compute Canada's Béluga computing cluster. The total computational cost for the 5.75μs of simulation time was around 40 core-years.

From the MD trajectories, we computed Dynamical Signatures by taking the mean-square fluctuations at the three atomic positions on which beads are placed for the ENMs in the present study: the P, C1′ and C2 atoms. This filtering ensures better comparison between

the MD trajectories and ENCoM Dynamical Signatures, in addition to providing a flexibility measure for each of the phosphate, sugar and base groups, aruguably the logical subdivision of a nucleotide into more or less rigid groups.

Because only 16 variants were simulated, training linear regression models on the full Dynamical Signatures would lead to severe overfitting since there are 257 positions in the signature. However, to see if the flexibility profiles from the MD simulations better capture dynamical patterns necessary for miR-125a maturation, we plotted the measured maturation efficiency against the euclidean distance between the WT dynamical signature and every mutant signature. Since there are three replicate trajectories per variant, we take the mean euclidean distance from the 9 pairwise distances (3 mutants X 3 WT).

Fig. A reports the correlation between the distance to the WT signature and the measured maturation efficiency. The expected correlation is negative: the further the dynamical profile is from the WT, the lower the maturation efficiency, giving rise to a negative correlation, as reported by Dallaire and coworkers [5]. Surprisingly, the MD signature leads to a very weak positive correlation. While the ENCoM EntroSigs do not reach statistical significance at R=-0.27, the correlation is of the expected sign. None of the four correlations reported reach statistical significance, however ENCoM EntroSigs perform better than the MD MSF.

We thus conclude that at the simulated timescales, MD simulations do not provide useful information over the ENCoM conformational space. Perhaps longer timescales would lead to better performance, however let us remind that the goal of our novel approach is the high-throughput computational prediction of variant effects. Since the computational cost of one ENCoM Entropic Signature is around 3 seconds on a single CPU, it represents a speedup of $8.8 * 10^6$ over the MD approach at the timescales presented here, namely slightly above 100ns per trajectory. The MD trajectories, converted to PDB format with the medoid frame from each 1ns window, are available online.

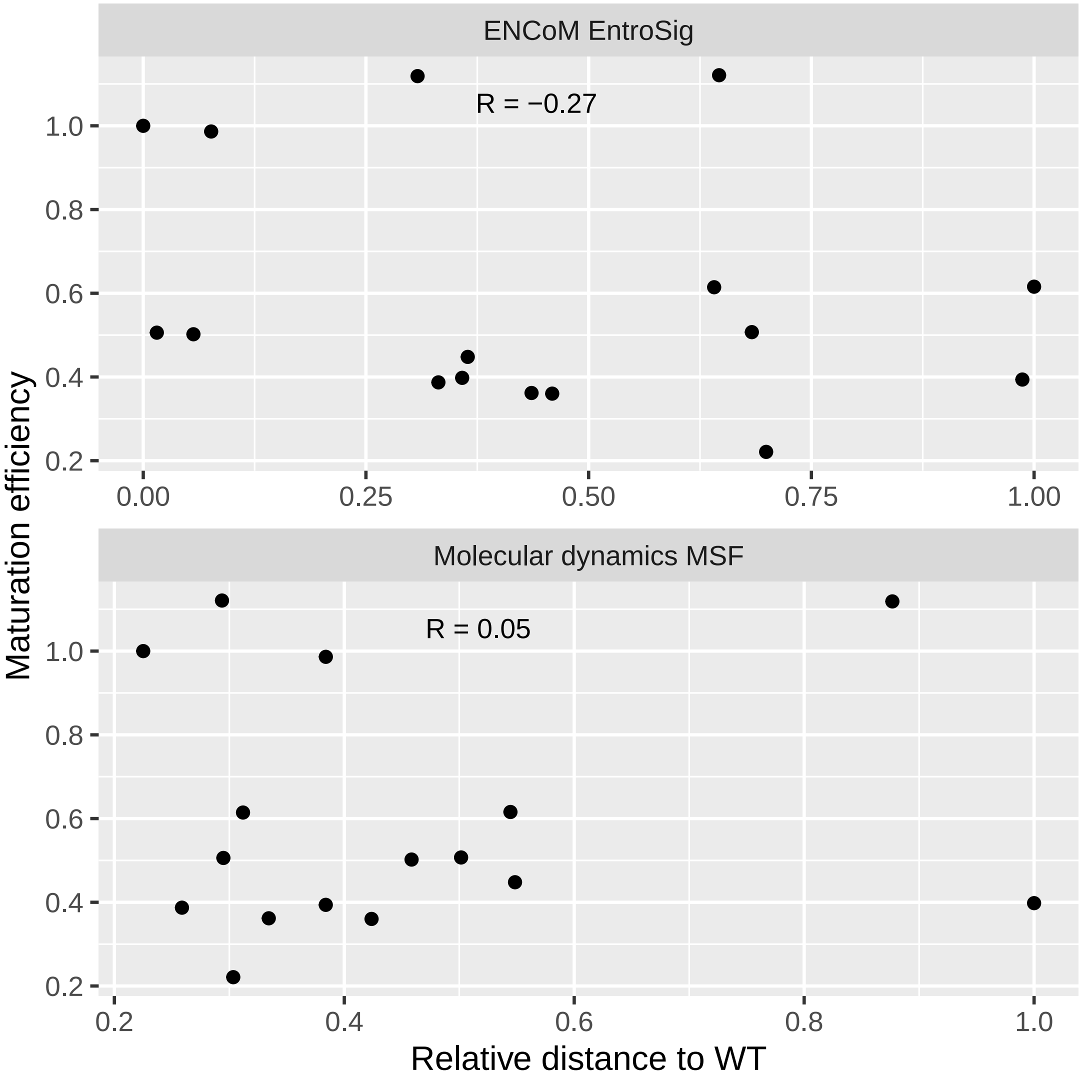

Fig. A: Correlation between miR-125a maturation efficiency and Dynamical Signature distance to WT for both ENCoM and MD simulations. The average distance for all 9 possibilities is reported for the MD simulations (3 replicates for the variant, 3 replicates for WT). The mean-square fluctuation was computed at P, C1′ and C2 atoms as the square of the RMSF computed with GROMACS. The ENCoM Entropic Signature was computed at $\beta = e^{0.25}$, the value selected from the B-factors benchmark. The Pearson linear correlation coefficients reported do not reach statistical significance.

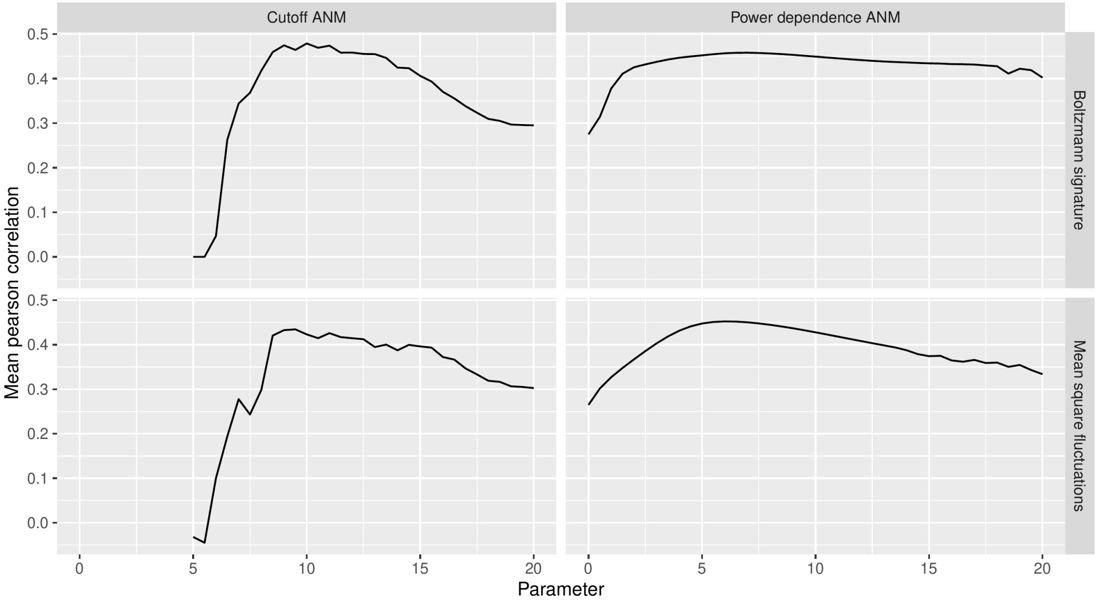

Fig. B: Cut-ANM and PD-ANM parameter sweep for the B-factors benchmark. Parameters are interaction distance cutoff (in Å) for Cut-ANM and power dependence for PD-ANM. Both are varied in increments of 0.5 from their minimum practical value up to 20.

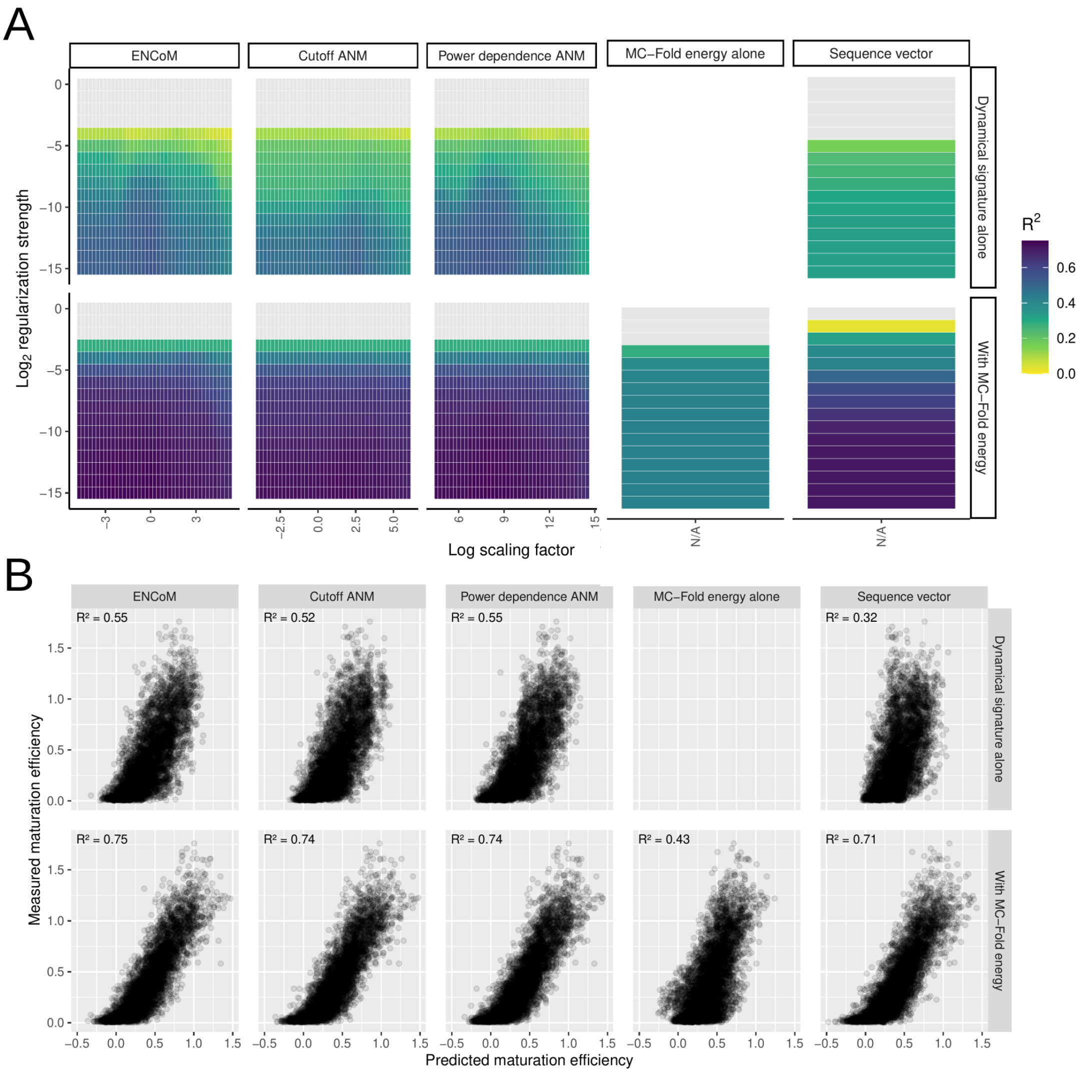

Fig. C: Performance of multiple linear regression on an 80-20 train-test split. A) Predictive R2 for each model alone or in combination with the MC-Fold enthalpy of folding. For the three ENMs, scaling factors for the dynamical signature were explored around the value which gave the best respective performance in the B-factors benchmark. Predictive R2 values below 0 are shown in gray. B) Performance on the test set for the best combination of parameters for every model alone or in tandem with MC-Fold.

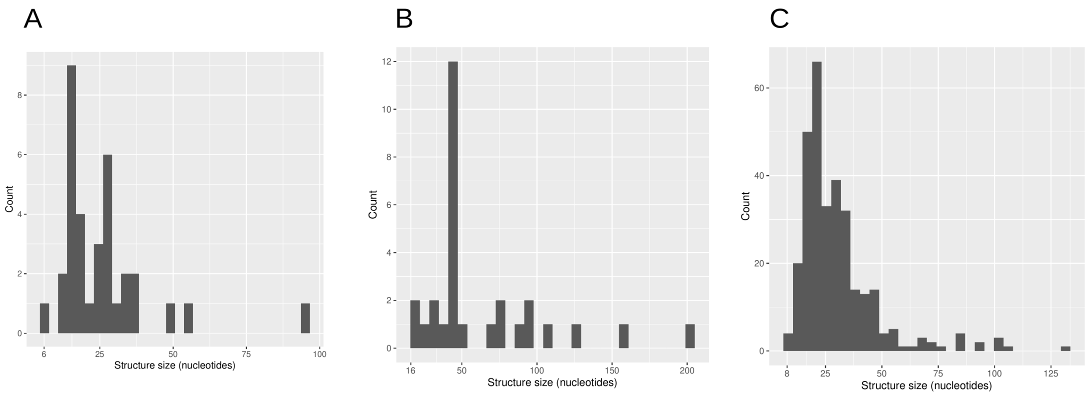

Fig. D: Size distributions of the sequence clusters from the 3 benchmarks. A) B-factors benchmark. B) Conformational change from X-ray structures benchmark. C) NMR ensemble structural variance benchmark.

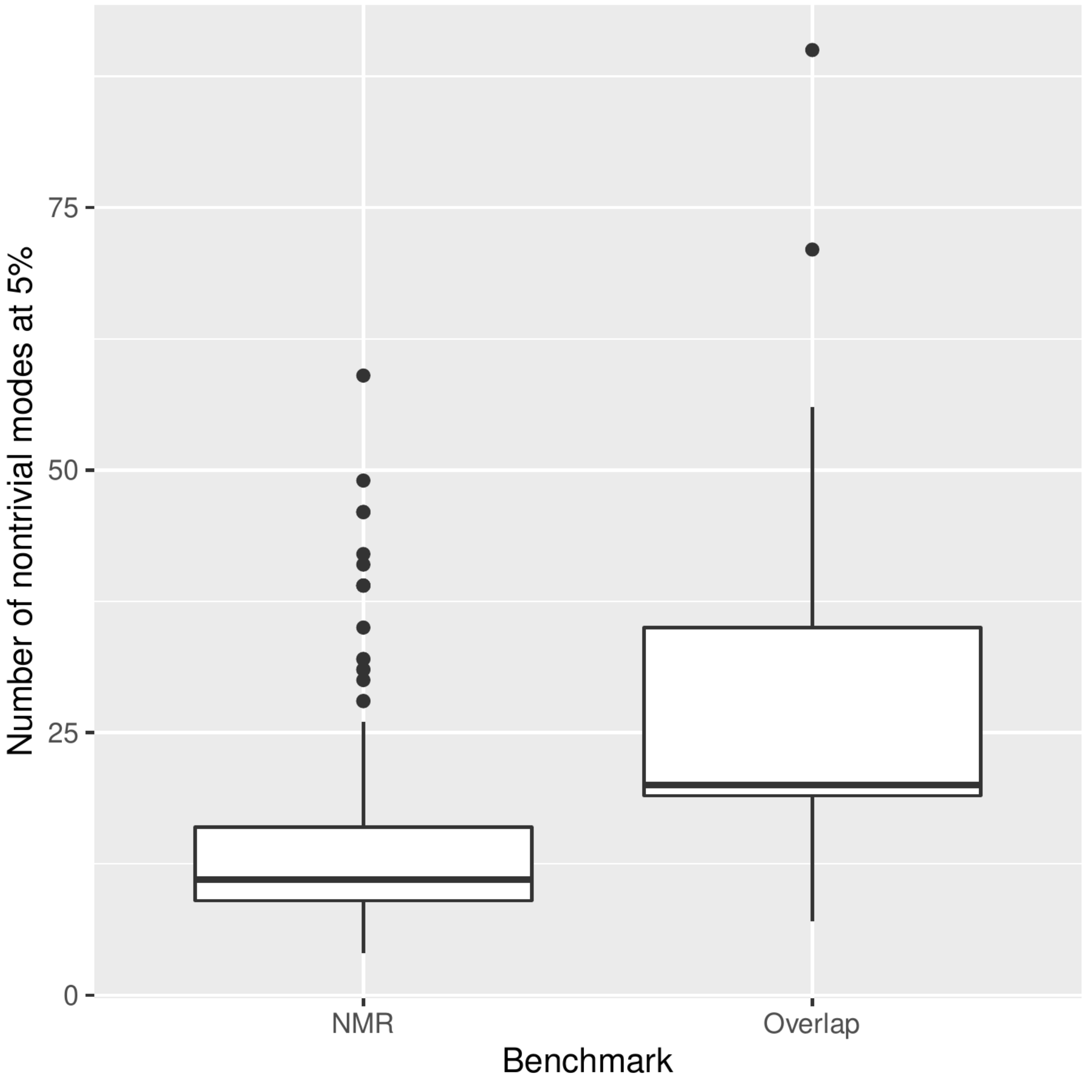

Fig. E: Number of nontrivial normal modes at 5% for the conformational change (overlap) and NMR ensemble variance benchmarks.

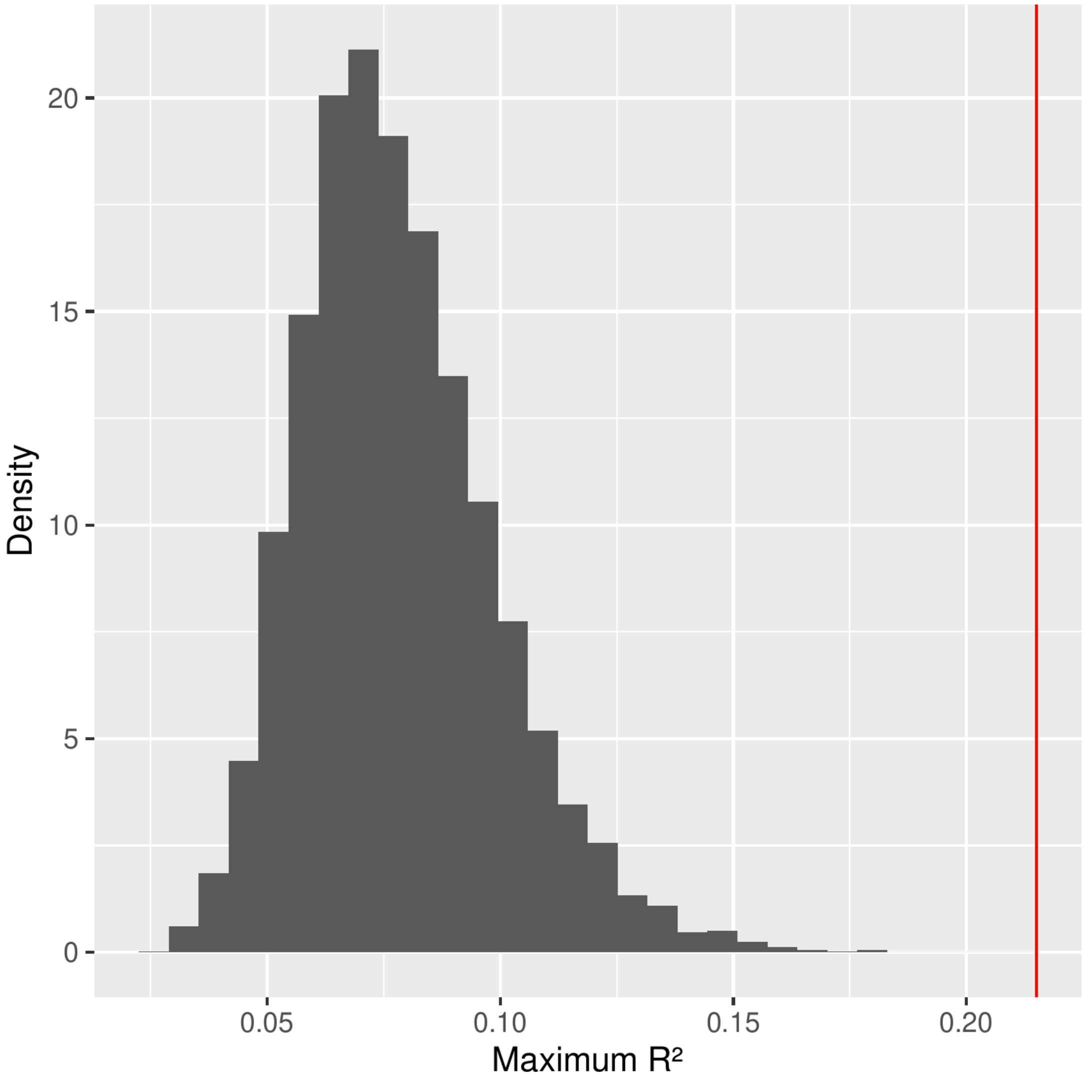

Fig. F: Simulation of predictive R-squared from combinations of MC-Fold enthalpy with random noise. Each of the 10 000 iterations represents the best predictive R2 value from 656 samplings of 116 values from a gaussian distribution centered around the training set mean and with a standard deviation two times smaller. The noise is then averaged at each sampling with the MC-Fold predictions and predictive R2 is computed. The red line shows the R2 of the ENCoM-MC-Fold combination on the hard benchmark (0.215).

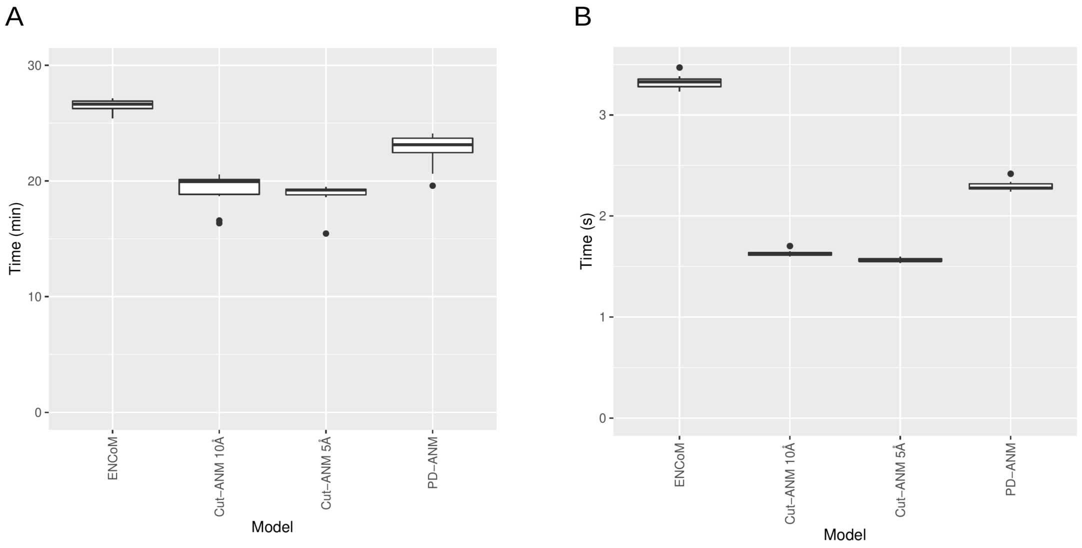

Fig. G: Computational cost of the ENMs on the Thermus thermophilus 30S ribosomal subunit and on miR-125a. A) Thermus thermophilus 30S ribosomal subunit. B) MiR-125a.

Table H: Atom type assignation of the four standard nucleotides. The atom types assigned are the ones from Sobolev and coworkers, divided in eight classes: hydrophilic, acceptor, donor, hydrophobic, aromatic, neutral, neutral-donor and neutral-acceptor.

Atom name	Atom type
Nucleobase atoms	
C2-C6, C8	Aromatic
N2, N4, N6	Donor
N3, N7	Acceptor
N9	Neutral
Sugar atoms	
C1′, C3′-C5′	Neutral-acceptor
C2′	Neutral
O4′	Acceptor
O2′	Hydrophilic
Phosphate atoms	
P	Neutral-acceptor
OP1-OP3, O5′, O3′	Acceptor
Specific atom types	
Adenine	
N1	Acceptor
Guanine	
N1	Donor
Cytidine	
N1	Neutral
N5	Acceptor
O2	Acceptor
Uridine	
O2	Acceptor
O4	Acceptor

Table I: PDB codes of the structures used for the three benchmarks.

B-factors prediction									
157D	1CSL	1DQH	1I9X	1KD5	1MSY	1OSU	1Q9A	1RNA	1RXB
1ZEV	255D	259D	280D	2OE6	2V6W	2V7R	2VUQ	2XSL	353D
3GM7	3SZX	402D	405D	413D	433D	438D	472D	480D	483D
4C40	4E59	4U37	4U38	5C5W	5NXT	5VGW	7EAG		
Conformational change prediction									
1HR2	1J7T	1L8V	1LC4	1MWL	1NLC	1O3Z	1O9M	1XP7	1XPE
1XPF	1Y3S	1YRJ	1YXP	1ZCI	1ZX7	1ZZ5	2A04	2B8R	2B8S
2BE0	2BEE	2ESJ	2ET3	2ET4	2ET5	2ET8	2F4S	2F4T	2F4U
2FCX	2FCY	2FCZ	2FD0	2FQN	2G5K	2GPM	2NOK	2O3W	2O3X
2OIJ	2OIY	2OJ0	2PN4	2PWT	2QEK	3BNL	3BNQ	3BNR	3BNS
3C44	3CW5	3CW6	3DVV	3FAR	3GCA	3L0U	3OWI	3OWW	3OWZ
3OX0	3OXB	3OXD	3OXE	3OXJ	3OXM	3TD0	3TD1	3WRU	439D
462D	4F8U	4F8V	4GPY	4K31	4K32	4L81	4MSB	4MSR	4OQU
4P20	4P3S	4P3T	4P3U	4P43	4PDQ	4RZD	4TZX	4TZY	4XNR
4Y1J	5E54	5L4O	5SWD	5SWE	5TDK	5UZA	5XZ1	5ZEG	5ZEI
5ZEJ	5ZEM	6BJX	6C8D	6CAB	6CB3	6D8L	6D8M	6D8O	6DLR
6DLS	6DNR	6E80	6E81	6E82	6E84	6JBG	6N5K	6N5O	6VUH
6VWT	6VWV	6WY3	6XKN	6XKO	6Y3G	6Z18	7EOI	7EOK	
NMR ensemble variance prediction									
17RA	1A3M	1A51	1A60	1A9L	1AFX	1ANR	1AQO	1ATO	1ATV
1ATW	1B36	1BGZ	1BN0	1BVJ	1CQL	1D0U	1E4P	1E95	1EBQ
1EBR	1EBS	1ELH	1ESY	1F5G	1F5H	1F7F	1F7G	1F84	1F85
1FHK	1FYO	1GUC	1HLX	1HWQ	1I3X	1IDV	1IE1	1IE2	1IKD
1JO7	1JOX	1JP0	1JTJ	1JU1	1JU7	1JUR	1K4A	1K4B	1K5I
1K6G	1K6H	1KKA	1KKS	1LC6	1LDZ	1LMV	1M5L	1M82	1MFJ
1MFK	1MFY	1MNX	1MT4	1N66	1N8X	1NA2	1NBR	1NC0	1OQ0
1OSW	1OW9	1P5M	1P5N	1P5O	1PJY	1Q75	1QC8	1QES	1QET
1QWA	1QWB	1R2P	1R7W	1R7Z	1RNG	1ROQ	1RRR	1S34	1S9S
1SCL	1SLP	1SY4	1SYZ	1T4X	1TBK	1TJZ	1TLR	1TXS	1U3K
1UUU	1VOP	1WKS	1XHP	1YLG	1YMO	1YN1	1YNC	1YNE	1YNG
1YSV	1Z2J	1Z30	1Z31	1ZC5	1ZIF	1ZIG	1ZIH	28SP	2ADT
2AHT	2B7G	2D17	2D18	2D19	2ES5	2EUY	2EVY	2F4X	2F87
2F88	2FDT	2FEY	2G1W	2GIO	2GIP	2GM0	2GRW	2GV3	2GV4
2GVO	2H49	2HEM	2HNS	2HUA	2IRN	2IRO	2IXY	2IXZ	2JR4
2JSE	2JTP	2JWV	2JXQ	2JXS	2JXV	2JYF	2JYH	2JYJ	2JYM
2K3Z	2K41	2K5Z	2K65	2K66	2K95	2K96	2KBP	2KD8	2KE6
2KEZ	2KF0	2KHY	2KOC	2KPC	2KPD	2KPV	2KRL	2KRP	2KRQ
2KUR	2KUU	2KUW	2KVN	2KXZ	2KY0	2KY1	2KY2	2KYD	2KZL
2L1F	2L2J	2L3E	2L5Z	2L6I	2L8F	2LAC	2LBJ	2LBK	2LBL
2LC8	2LDL	2LDT	2LHP	2LJJ	2LK3	2LP9	2LPA	2LPS	2LPT
2LQZ	2LU0	2LUB	2LUN	2LV0	2LX1	2M12	2M18	2M21	2M22
2M23	2M24	2M4W	2M57	2M5U	2M8K	2MEQ	2MFD	2MI0	2MNC
2MQT	2MTJ	2MXJ	2MXK	2MXL	2N2O	2N2P	2N3Q	2N4L	2N6S
2N6T	2N6W	2N6X	2N7M	2N7X	2N8V	2NBX	2NBY	2NBZ	2NC0
2NC1	2NCI	2O33	2OJ7	2OJ8	2P89	2PCV	2PCW	2QH2	2QH3
2QH4	2RLU	2RN1	2RPT	2RQJ	2RVO	2XEB	2Y95	3PHP	4A4S
4A4T	4A4U	5A17	5A18	5IEM	5KH8	5KMZ	5KQE	5LSN	5N5C
5UF3	5UZT	5V16	5V17	5V2R	5VH7	5VH8	5WQ1	6AAS	6BG9
6BY4	6BY5	6GE1	6HYK	6K84	6MXQ	6N8F	6N8I	6NOA	6PK9
6U79	6VA1	6VAR	6VZC	6W3M	6XWJ	6XWW	6XXA	6XXB	7DD4
7JU1	7K4L	7LVA							

Table J: Performance on individual structures for the B-factors correlation benchmark.

PDB code	Cluster index	ENCoM BZ	ENCoM MSF	Cut-ANM BZ	Cut-ANM MSF	PD-ANM BZ	PD-ANM MSF
472D	1	0.49	0.49	0.55	0.52	0.55	0.55
4U37	2	0.78	0.79	0.71	0.68	0.65	0.66
433D	3	0.59	0.60	0.45	0.57	0.42	0.50
405D	4	0.52	0.45	0.60	0.46	0.61	0.56
1CSL	5	0.40	0.36	0.32	0.17	0.28	0.35
1Q9A	6	0.44	0.37	0.65	0.38	0.56	0.49
480D	6	0.65	0.65	0.76	0.66	0.70	0.67
483D	6	0.39	0.33	0.56	0.34	0.50	0.44
5C5W	7	0.69	0.77	0.45	0.77	0.47	0.77
2VUQ	8	0.83	0.80	0.78	0.72	0.71	0.73
1I9X	9	0.31	0.31	0.19	0.07	0.12	0.08
1RNA	10	0.55	0.51	0.58	0.41	0.57	0.52
413D	11	0.68	0.70	0.66	0.80	0.68	0.74
280D	12	0.21	0.06	0.31	0.26	0.31	0.05
157D	13	0.25	0.17	0.56	0.46	0.54	0.51
3SZX	14	0.35	0.38	0.28	0.41	0.29	0.36
353D	15	-0.06	-0.09	0.15	-0.05	0.12	0.12
255D	16	0.68	0.64	0.70	0.77	0.71	0.71
1ZEV	17	0.31	0.25	0.10	-0.05	0.04	0.01
3GM7	17	0.43	0.35	0.31	0.34	0.32	0.31
1KD5	18	0.18	0.16	0.44	0.19	0.38	0.25
5VGW	19	0.09	0.03	0.37	0.35	0.31	0.18
4C40	20	0.62	0.50	0.70	0.44	0.75	0.81
402D	21	0.42	0.43	0.40	0.44	0.42	0.43
1RXB	22	0.59	0.60	0.64	0.65	0.60	0.63
259D	22	0.57	0.58	0.59	0.63	0.54	0.58
2V6W	23	0.71	0.73	0.73	0.66	0.73	0.74
2XSL	24	0.45	0.47	0.50	0.48	0.53	0.50
1DQH	25	0.44	0.46	0.58	0.54	0.54	0.52
7EAG	26	-0.08	-0.20	0.09	-0.09	0.01	-0.09
438D	27	0.47	0.42	0.54	0.46	0.52	0.56
5NXT	28	-0.01	-0.01	0.13	0.13	0.11	0.04
1MSY	29	0.63	0.68	0.65	0.76	0.63	0.64
2V7R	30	0.82	0.78	0.79	0.72	0.71	0.74
4U38	31	0.28	0.23	0.29	0.20	0.32	0.24
2OE6	32	0.48	0.42	0.44	0.39	0.41	0.43
1OSU	33	0.20	0.17	0.23	0.24	0.32	0.29
4E59	34	0.58	0.59	0.64	0.60	0.58	0.58

Table K: Performance on individual pairs of conformations for the X-ray conformational change benchmark.

Input PDB code	Target PDB code	RMSD	Cluster index	ENCoM	Cut-ANM	PD-ANM
1HR2	1L8V	2.26	1	0.66	0.54	0.57
1HR2	6BJX	2.21	1	0.52	0.44	0.45
1HR2	6D8L	2.22	1	0.52	0.45	0.46
1HR2	6D8M	2.15	1	0.56	0.48	0.52
1HR2	6D8O	2.01	1	0.56	0.46	0.49
1L8V	1HR2	2.26	1	0.58	0.55	0.55
1L8V	6BJX	2.24	1	0.58	0.53	0.57
1L8V	6D8L	2.25	1	0.58	0.53	0.56
1L8V	6D8M	2.14	1	0.63	0.58	0.59
1L8V	6D8O	2.14	1	0.64	0.57	0.58
6BJX	1HR2	2.21	1	0.50	0.42	0.45
6BJX	1L8V	2.24	1	0.60	0.51	0.53
6D8L	1HR2	2.22	1	0.50	0.43	0.44
6D8L	1L8V	2.25	1	0.58	0.50	0.52
6D8M	1HR2	2.15	1	0.53	0.46	0.47
6D8M	1L8V	2.14	1	0.63	0.57	0.57
6D8O	1HR2	2.01	1	0.53	0.46	0.46
6D8O	1L8V	2.14	1	0.64	0.55	0.57
1J7T	1O9M	2.02	2	0.63	0.66	0.67
1J7T	2ET8	2.02	2	0.59	0.57	0.60
1J7T	3BNL	2.90	2	0.77	0.80	0.81
1J7T	4F8U	2.36	2	0.92	0.90	0.92
1LC4	3BNL	2.96	2	0.78	0.84	0.83
1LC4	4F8U	2.07	2	0.91	0.92	0.92
1MWL	1O9M	2.14	2	0.63	0.66	0.66
1MWL	2ET8	2.24	2	0.63	0.61	0.63
1MWL	3BNL	3.32	2	0.80	0.83	0.85
1MWL	4F8U	2.25	2	0.79	0.75	0.77
1O9M	1J7T	2.02	2	0.63	0.65	0.65
1O9M	1MWL	2.14	2	0.64	0.65	0.69
1O9M	1YRJ	2.43	2	0.68	0.67	0.69
1O9M	2BE0	2.06	2	0.64	0.63	0.68
1O9M	2BEE	2.05	2	0.64	0.63	0.68
1O9M	2ET3	2.31	2	0.70	0.72	0.74
1O9M	2F4S	2.64	2	0.47	0.57	0.55
1O9M	2F4T	2.00	2	0.32	0.24	0.22
1O9M	2F4U	2.65	2	0.49	0.58	0.57
1O9M	2O3X	2.55	2	0.43	0.53	0.52
1O9M	2PWT	2.18	2	0.71	0.73	0.75
1O9M	3BNL	2.91	2	0.72	0.75	0.76
1O9M	4F8U	2.59	2	0.73	0.70	0.72
1O9M	4F8V	2.28	2	0.67	0.66	0.68
1YRJ	1O9M	2.43	2	0.69	0.63	0.60
1YRJ	2ET8	2.44	2	0.68	0.64	0.63
1YRJ	2F4S	2.51	2	0.68	0.63	0.65
1YRJ	2F4T	2.47	2	0.67	0.62	0.62
1YRJ	2F4U	2.23	2	0.62	0.59	0.60
1YRJ	2O3X	2.38	2	0.67	0.60	0.63
1YRJ	2PWT	2.04	2	0.84	0.76	0.76

Table K (continued)						
Input PDB code	Target PDB code	RMSD	Cluster index	ENCoM	Cut-ANM	PD-ANM
1YRJ	3BNL	3.44	2	0.79	0.77	0.77
1YRJ	4F8U	2.81	2	0.88	0.78	0.78
1YRJ	4F8V	2.02	2	0.85	0.80	0.82
2BE0	1O9M	2.06	2	0.66	0.65	0.67
2BE0	2ET8	2.23	2	0.70	0.64	0.67
2BE0	2F4S	2.33	2	0.71	0.69	0.71
2BE0	2F4U	2.02	2	0.66	0.67	0.69
2BE0	2O3X	2.16	2	0.67	0.65	0.66
2BE0	3BNL	3.15	2	0.84	0.86	0.86
2BE0	4F8U	2.50	2	0.82	0.80	0.82
2BEE	1O9M	2.05	2	0.66	0.64	0.66
2BEE	2ET8	2.21	2	0.69	0.63	0.66
2BEE	2F4S	2.32	2	0.71	0.70	0.70
2BEE	2F4U	2.02	2	0.66	0.66	0.68
2BEE	2O3X	2.16	2	0.67	0.66	0.65
2BEE	3BNL	3.15	2	0.84	0.85	0.86
2BEE	4F8U	2.49	2	0.91	0.89	0.91
2ESJ	2F4S	2.02	2	0.58	0.53	0.58
2ESJ	3BNL	2.83	2	0.78	0.81	0.82
2ESJ	4F8U	2.44	2	0.81	0.79	0.80
2ET3	1O9M	2.31	2	0.71	0.71	0.72
2ET3	2ET8	2.35	2	0.70	0.67	0.69
2ET3	2F4S	2.01	2	0.62	0.55	0.56
2ET3	2F4T	2.13	2	0.65	0.59	0.61
2ET3	3BNL	3.24	2	0.80	0.82	0.82
2ET3	4F8U	2.50	2	0.92	0.88	0.89
2ET4	3BNL	2.88	2	0.74	0.78	0.79
2ET4	4F8U	2.11	2	0.79	0.79	0.79
2ET5	2ET8	2.05	2	0.60	0.54	0.60
2ET5	3BNL	2.90	2	0.75	0.77	0.79
2ET5	4F8U	2.46	2	0.85	0.83	0.85
2ET8	1J7T	2.02	2	0.60	0.57	0.57
2ET8	1MWL	2.24	2	0.66	0.64	0.64
2ET8	1YRJ	2.44	2	0.63	0.61	0.62
2ET8	2BE0	2.23	2	0.69	0.66	0.66
2ET8	2BEE	2.21	2	0.69	0.65	0.65
2ET8	2ET3	2.35	2	0.70	0.70	0.71
2ET8	2ET5	2.05	2	0.59	0.55	0.54
2ET8	2F4S	2.53	2	0.36	0.44	0.45
2ET8	2F4T	2.15	2	0.34	0.23	0.23
2ET8	2F4U	2.57	2	0.43	0.51	0.51
2ET8	2O3X	2.47	2	0.33	0.40	0.41
2ET8	2PWT	2.25	2	0.72	0.70	0.71
2ET8	3BNL	2.78	2	0.69	0.73	0.73
2ET8	4F8U	2.61	2	0.69	0.65	0.66
2ET8	4F8V	2.19	2	0.62	0.59	0.59
2F4S	1O9M	2.64	2	0.49	0.53	0.54
2F4S	1YRJ	2.51	2	0.60	0.62	0.62
2F4S	2BE0	2.33	2	0.71	0.66	0.67
2F4S	2BEE	2.32	2	0.71	0.66	0.67

Input PDB code	Target PDB code	RMSD	Cluster index	ENCoM	Cut-ANM	PD-ANM
2F4S	2ESJ	2.02	2	0.58	0.59	0.61
2F4S	2ET3	2.01	2	0.63	0.60	0.60
2F4S	2ET8	2.53	2	0.37	0.46	0.47
2F4S	2F4T	2.32	2	0.46	0.41	0.43
2F4S	2PWT	2.28	2	0.70	0.67	0.68
2F4S	3BNL	3.11	2	0.64	0.68	0.69
2F4S	4F8U	2.66	2	0.76	0.73	0.74
2F4T	1O9M	2.00	2	0.49	0.48	0.51
2F4T	1YRJ	2.47	2	0.50	0.47	0.48
2F4T	2ET3	2.13	2	0.78	0.81	0.78
2F4T	2ET8	2.15	2	0.49	0.44	0.46
2F4T	2F4S	2.32	2	0.52	0.59	0.60
2F4T	2F4U	2.35	2	0.56	0.61	0.61
2F4T	2O3X	2.23	2	0.48	0.55	0.57
2F4T	3BNL	3.00	2	0.60	0.65	0.64
2F4T	4F8U	2.39	2	0.79	0.76	0.79
2F4T	4F8V	2.17	2	0.79	0.73	0.73
2F4U	1O9M	2.65	2	0.50	0.57	0.56
2F4U	1YRJ	2.23	2	0.54	0.55	0.54
2F4U	2BE0	2.02	2	0.65	0.65	0.68
2F4U	2BEE	2.02	2	0.65	0.65	0.67
2F4U	2ET8	2.57	2	0.43	0.50	0.50
2F4U	2F4T	2.35	2	0.42	0.43	0.42
2F4U	2PWT	2.06	2	0.60	0.58	0.61
2F4U	3BNL	2.81	2	0.74	0.78	0.78
2F4U	4F8U	2.68	2	0.81	0.79	0.81
2O3X	1O9M	2.55	2	0.44	0.53	0.55
2O3X	1YRJ	2.38	2	0.57	0.62	0.62
2O3X	2BE0	2.16	2	0.67	0.65	0.66
2O3X	2BEE	2.16	2	0.66	0.64	0.66
2O3X	2ET8	2.47	2	0.34	0.46	0.47
2O3X	2F4T	2.23	2	0.37	0.37	0.39
2O3X	2PWT	2.13	2	0.66	0.66	0.68
2O3X	3BNL	3.12	2	0.66	0.69	0.69
2O3X	4F8U	2.57	2	0.77	0.77	0.78
2PWT	1O9M	2.18	2	0.71	0.72	0.73
2PWT	1YRJ	2.04	2	0.70	0.71	0.71
2PWT	2ET8	2.25	2	0.71	0.65	0.68
2PWT	2F4S	2.28	2	0.69	0.72	0.72
2PWT	2F4U	2.06	2	0.60	0.60	0.62
2PWT	2O3X	2.13	2	0.65	0.69	0.68
2PWT	3BNL	3.17	2	0.80	0.81	0.82
2PWT	4F8U	2.68	2	0.93	0.91	0.91
3BNL	1J7T	2.90	2	0.78	0.68	0.72
3BNL	1LC4	2.96	2	0.80	0.64	0.71
3BNL	1MWL	3.32	2	0.82	0.69	0.76
3BNL	1O9M	2.91	2	0.74	0.62	0.68
3BNL	1YRJ	3.44	2	0.78	0.73	0.75
3BNL	2BE0	3.15	2	0.85	0.72	0.78
3BNL	2BEE	3.15	2	0.84	0.71	0.77

Table K (continued)

Table K (continued)						
Input PDB code	Target PDB code	RMSD	Cluster index	ENCoM	Cut-ANM	PD-ANM
3BNL	2ESJ	2.83	2	0.80	0.69	0.73
3BNL	2ET3	3.24	2	0.81	0.74	0.77
3BNL	2ET4	2.88	2	0.75	0.61	0.68
3BNL	2ET5	2.90	2	0.76	0.63	0.68
3BNL	2ET8	2.78	2	0.71	0.60	0.65
3BNL	2F4S	3.11	2	0.66	0.56	0.65
3BNL	2F4T	3.00	2	0.62	0.52	0.59
3BNL	2F4U	2.81	2	0.75	0.63	0.69
3BNL	2O3X	3.12	2	0.67	0.56	0.65
3BNL	2PWT	3.17	2	0.81	0.68	0.73
3BNL	4F8U	4.09	2	0.88	0.82	0.85
3BNL	4F8V	3.15	2	0.77	0.66	0.68
3BNL	4P20	3.03	2	0.80	0.65	0.71
4F8U	1J7T	2.36	2	0.92	0.93	0.93
4F8U	1LC4	2.07	2	0.89	0.91	0.92
4F8U	1MWL	2.25	2	0.90	0.92	0.92
4F8U	1O9M	2.59	2	0.73	0.75	0.74
4F8U	1YRJ	2.81	2	0.84	0.83	0.82
4F8U	2BE0	2.50	2	0.89	0.91	0.91
4F8U	2BEE	2.49	2	0.89	0.91	0.91
4F8U	2ESJ	2.44	2	0.89	0.91	0.91
4F8U	2ET3	2.50	2	0.94	0.94	0.94
4F8U	2ET4	2.11	2	0.91	0.92	0.92
4F8U	2ET5	2.46	2	0.92	0.94	0.94
4F8U	2ET8	2.61	2	0.72	0.71	0.71
4F8U	2F4S	2.66	2	0.78	0.77	0.77
4F8U	2F4T	2.39	2	0.70	0.70	0.70
4F8U	2F4U	2.68	2	0.81	0.83	0.83
4F8U	2O3X	2.57	2	0.78	0.77	0.77
4F8U	2PWT	2.68	2	0.92	0.94	0.94
4F8U	3BNL	4.09	2	0.81	0.82	0.81
4F8U	4F8V	2.56	2	0.92	0.93	0.93
4F8U	4P20	2.09	2	0.91	0.93	0.93
4F8V	1O9M	2.28	2	0.66	0.70	0.74
4F8V	1YRJ	2.02	2	0.83	0.82	0.84
4F8V	2ET8	2.19	2	0.60	0.62	0.67
4F8V	2F4T	2.17	2	0.62	0.60	0.62
4F8V	3BNL	3.15	2	0.77	0.78	0.81
4F8V	4F8U	2.56	2	0.84	0.81	0.85
4P20	3BNL	3.03	2	0.78	0.81	0.82
4P20	4F8U	2.09	2	0.89	0.85	0.89
1NLC	1XP7	23.52	3	0.80	0.66	0.69
1NLC	1XPF	23.96	3	0.79	0.65	0.69
1NLC	1Y3S	23.66	3	0.80	0.66	0.70
1NLC	1YXP	23.70	3	0.80	0.66	0.69
1NLC	1ZCI	23.34	3	0.80	0.65	0.69
1NLC	2B8S	23.41	3	0.80	0.66	0.70
1NLC	2FCX	22.97	3	0.81	0.68	0.72
1NLC	2FCY	23.08	3	0.81	0.67	0.71
1NLC	2FCZ	22.94	3	0.81	0.67	0.71

Table K (continued)						
Input PDB code	Target PDB code	RMSD	Cluster index	ENCoM	Cut-ANM	PD-ANM
1NLC	2FD0	23.28	3	0.80	0.66	0.70
1NLC	2QEK	2.60	3	0.63	0.54	0.60
1NLC	3C44	2.44	3	0.74	0.64	0.68
1NLC	3DVV	2.76	3	0.73	0.63	0.65
1O3Z	1XP7	23.50	3	0.80	0.66	0.69
1O3Z	1XPF	23.93	3	0.79	0.65	0.68
1O3Z	1Y3S	23.64	3	0.80	0.66	0.69
1O3Z	1YXP	23.68	3	0.80	0.66	0.69
1O3Z	1ZCI	23.31	3	0.80	0.66	0.69
1O3Z	2B8S	23.38	3	0.80	0.66	0.69
1O3Z	2FCX	22.94	3	0.81	0.68	0.71
1O3Z	2FCY	23.06	3	0.81	0.67	0.71
1O3Z	2FCZ	22.91	3	0.81	0.67	0.71
1O3Z	2FD0	23.25	3	0.80	0.66	0.70
1O3Z	2QEK	2.64	3	0.64	0.57	0.63
1O3Z	3C44	2.33	3	0.72	0.62	0.65
1O3Z	3DVV	2.68	3	0.72	0.61	0.62
1XP7	1NLC	23.52	3	0.79	0.65	0.71
1XP7	1O3Z	23.50	3	0.79	0.65	0.71
1XP7	2QEK	23.49	3	0.79	0.67	0.72
1XP7	3C44	23.02	3	0.78	0.66	0.72
1XP7	3DVV	22.68	3	0.78	0.67	0.73
1XP7	462D	23.54	3	0.79	0.65	0.71
1XPF	1NLC	23.96	3	0.80	0.67	0.71
1XPF	1O3Z	23.93	3	0.80	0.67	0.71
1XPF	2QEK	23.93	3	0.80	0.69	0.72
1XPF	3C44	23.46	3	0.79	0.69	0.73
1XPF	3DVV	23.12	3	0.79	0.70	0.73
1XPF	462D	23.97	3	0.80	0.67	0.71
1Y3S	1NLC	23.66	3	0.79	0.66	0.72
1Y3S	1O3Z	23.64	3	0.79	0.66	0.72
1Y3S	2QEK	23.63	3	0.79	0.67	0.73
1Y3S	3C44	23.16	3	0.78	0.67	0.73
1Y3S	3DVV	22.81	3	0.78	0.68	0.74
1Y3S	462D	23.67	3	0.79	0.66	0.72
1YXP	1NLC	23.70	3	0.79	0.66	0.72
1YXP	1O3Z	23.68	3	0.79	0.66	0.72
1YXP	2QEK	23.67	3	0.79	0.67	0.73
1YXP	3C44	23.19	3	0.78	0.67	0.73
1YXP	3DVV	22.84	3	0.78	0.68	0.73
1YXP	462D	23.72	3	0.79	0.66	0.72
1ZCI	1NLC	23.34	3	0.83	0.61	0.71
1ZCI	1O3Z	23.31	3	0.83	0.61	0.71
1ZCI	2FCX	2.08	3	0.84	0.83	0.82
1ZCI	2QEK	23.36	3	0.82	0.62	0.72
1ZCI	3C44	22.85	3	0.83	0.62	0.73
1ZCI	3DVV	22.52	3	0.83	0.63	0.73
1ZCI	462D	23.35	3	0.83	0.61	0.71
2B8S	1NLC	23.41	3	0.79	0.65	0.70
2B8S	1O3Z	23.38	3	0.79	0.65	0.70

Table K (continued)						
Input PDB code	Target PDB code	RMSD	Cluster index	ENCoM	Cut-ANM	PD-ANM
2B8S	2QEK	23.38	3	0.79	0.67	0.71
2B8S	3C44	22.90	3	0.78	0.67	0.71
2B8S	3DVV	22.56	3	0.78	0.67	0.72
2B8S	462D	23.42	3	0.79	0.65	0.70
2FCX	1NLC	22.97	3	0.79	0.64	0.73
2FCX	1O3Z	22.94	3	0.79	0.64	0.73
2FCX	1ZCI	2.08	3	0.88	0.78	0.81
2FCX	2QEK	22.90	3	0.78	0.65	0.73
2FCX	3C44	22.46	3	0.78	0.66	0.74
2FCX	3DVV	22.10	3	0.78	0.66	0.74
2FCX	462D	22.98	3	0.79	0.64	0.73
2FCY	1NLC	23.08	3	0.80	0.63	0.70
2FCY	1O3Z	23.06	3	0.79	0.63	0.69
2FCY	2QEK	23.03	3	0.80	0.64	0.70
2FCY	3C44	22.55	3	0.79	0.64	0.70
2FCY	3DVV	22.22	3	0.79	0.65	0.71
2FCY	462D	23.10	3	0.80	0.63	0.69
2FCZ	1NLC	22.94	3	0.79	0.63	0.73
2FCZ	1O3Z	22.91	3	0.79	0.63	0.73
2FCZ	2QEK	22.91	3	0.79	0.64	0.73
2FCZ	3C44	22.41	3	0.79	0.64	0.73
2FCZ	3DVV	22.06	3	0.79	0.65	0.73
2FCZ	462D	22.94	3	0.79	0.63	0.72
2FD0	1NLC	23.28	3	0.80	0.65	0.71
2FD0	1O3Z	23.25	3	0.80	0.65	0.71
2FD0	2QEK	23.26	3	0.80	0.66	0.71
2FD0	3C44	22.76	3	0.80	0.66	0.72
2FD0	3DVV	22.42	3	0.80	0.67	0.72
2FD0	462D	23.28	3	0.80	0.65	0.71
2QEK	1NLC	2.60	3	0.67	0.61	0.59
2QEK	1O3Z	2.64	3	0.68	0.63	0.60
2QEK	1XP7	23.49	3	0.80	0.64	0.69
2QEK	1XPF	23.93	3	0.79	0.64	0.69
2QEK	1Y3S	23.63	3	0.80	0.65	0.70
2QEK	1YXP	23.67	3	0.80	0.65	0.70
2QEK	1ZCI	23.36	3	0.80	0.64	0.69
2QEK	2B8S	23.38	3	0.80	0.65	0.70
2QEK	2FCX	22.90	3	0.81	0.67	0.72
2QEK	2FCY	23.03	3	0.80	0.66	0.71
2QEK	2FCZ	22.91	3	0.81	0.66	0.71
2QEK	2FD0	23.26	3	0.80	0.65	0.70
2QEK	3C44	3.19	3	0.79	0.78	0.79
2QEK	3DVV	3.17	3	0.79	0.78	0.79
2QEK	462D	2.93	3	0.75	0.69	0.67
3C44	1NLC	2.44	3	0.70	0.59	0.61
3C44	1O3Z	2.33	3	0.68	0.56	0.58
3C44	1XP7	23.02	3	0.80	0.62	0.64
3C44	1XPF	23.46	3	0.79	0.61	0.63
3C44	1Y3S	23.16	3	0.80	0.62	0.64
3C44	1YXP	23.19	3	0.80	0.62	0.64

Table K (continued)						
Input PDB code	Target PDB code	RMSD	Cluster index	ENCoM	Cut-ANM	PD-ANM
3C44	1ZCI	22.85	3	0.80	0.62	0.64
3C44	2B8S	22.90	3	0.80	0.62	0.65
3C44	2FCX	22.46	3	0.81	0.64	0.66
3C44	2FCY	22.55	3	0.80	0.63	0.66
3C44	2FCZ	22.41	3	0.81	0.64	0.66
3C44	2FD0	22.76	3	0.80	0.63	0.64
3C44	2QEK	3.19	3	0.67	0.63	0.62
3C44	462D	2.59	3	0.73	0.67	0.67
3DVV	1NLC	2.76	3	0.69	0.61	0.66
3DVV	1O3Z	2.68	3	0.68	0.59	0.65
3DVV	1XP7	22.68	3	0.80	0.57	0.62
3DVV	1XPF	23.12	3	0.79	0.56	0.61
3DVV	1Y3S	22.81	3	0.81	0.57	0.62
3DVV	1YXP	22.84	3	0.81	0.57	0.62
3DVV	1ZCI	22.52	3	0.80	0.57	0.62
3DVV	2B8S	22.56	3	0.80	0.58	0.62
3DVV	2FCX	22.10	3	0.81	0.59	0.64
3DVV	2FCY	22.22	3	0.81	0.58	0.63
3DVV	2FCZ	22.06	3	0.81	0.59	0.64
3DVV	2FD0	22.42	3	0.80	0.58	0.63
3DVV	2QEK	3.17	3	0.64	0.62	0.64
3DVV	462D	2.65	3	0.68	0.60	0.64
462D	1XP7	23.54	3	0.80	0.66	0.69
462D	1XPF	23.97	3	0.79	0.66	0.68
462D	1Y3S	23.67	3	0.81	0.66	0.69
462D	1YXP	23.72	3	0.81	0.66	0.68
462D	1ZCI	23.35	3	0.80	0.66	0.69
462D	2B8S	23.42	3	0.80	0.67	0.69
462D	2FCX	22.98	3	0.81	0.68	0.70
462D	2FCY	23.10	3	0.81	0.67	0.70
462D	2FCZ	22.94	3	0.81	0.68	0.70
462D	2FD0	23.28	3	0.80	0.67	0.69
462D	2QEK	2.93	3	0.72	0.63	0.69
462D	3C44	2.59	3	0.77	0.72	0.72
462D	3DVV	2.65	3	0.72	0.60	0.62
1XPE	2OIJ	22.73	4	0.78	0.65	0.71
1XPE	2OIY	22.66	4	0.78	0.65	0.71
1XPE	2OJ0	22.97	4	0.78	0.65	0.71
1XPE	3FAR	22.76	4	0.78	0.65	0.71
2B8R	2OIJ	22.79	4	0.79	0.66	0.71
2B8R	2OIY	22.72	4	0.79	0.66	0.71
2B8R	2OJ0	23.03	4	0.78	0.66	0.71
2B8R	3FAR	22.82	4	0.79	0.66	0.71
2OIJ	1XPE	22.73	4	0.82	0.68	0.68
2OIJ	2B8R	22.79	4	0.82	0.68	0.68
2OIY	1XPE	22.66	4	0.82	0.66	0.68
2OIY	2B8R	22.72	4	0.82	0.66	0.68
2OJ0	1XPE	22.97	4	0.82	0.68	0.70
2OJ0	2B8R	23.03	4	0.82	0.68	0.70
3FAR	1XPE	22.76	4	0.82	0.67	0.70

Table K (continued)						
Input PDB code	Target PDB code	RMSD	Cluster index	ENCoM	Cut-ANM	PD-ANM
3FAR	2B8R	22.82	4	0.82	0.67	0.69
1ZX7	1ZZ5	2.15	5	0.87	0.81	0.82
1ZX7	2A04	2.17	5	0.86	0.81	0.83
1ZZ5	1ZX7	2.15	5	0.88	0.83	0.83
2A04	1ZX7	2.17	5	0.89	0.82	0.83
2FQN	2G5K	3.06	6	0.60	0.52	0.53
2FQN	5XZ1	3.37	6	0.66	0.62	0.62
2G5K	2FQN	3.06	6	0.57	0.60	0.60
2G5K	2O3W	2.86	6	0.51	0.54	0.54
2G5K	5XZ1	3.97	6	0.63	0.62	0.64
2O3W	2G5K	2.86	6	0.51	0.45	0.46
2O3W	5XZ1	3.05	6	0.59	0.53	0.54
5XZ1	2FQN	3.37	6	0.73	0.66	0.72
5XZ1	2G5K	3.97	6	0.65	0.59	0.62
5XZ1	2O3W	3.05	6	0.65	0.62	0.65
2GPM	439D	7.85	7	0.40	0.16	0.18
439D	2GPM	7.85	7	0.36	0.16	0.16
2NOK	2PN4	2.02	8	0.88	0.83	0.87
2PN4	2NOK	2.02	8	0.87	0.85	0.87
3BNQ	3BNR	5.86	9	0.74	0.66	0.68
3BNQ	3BNS	5.89	9	0.74	0.66	0.68
3BNR	3BNQ	5.86	9	0.73	0.67	0.69
3BNS	3BNQ	5.89	9	0.73	0.66	0.68
3CW5	5L4O	2.89	10	0.87	0.79	0.82
3CW6	5L4O	2.89	10	0.87	0.80	0.82
5L4O	3CW5	2.89	10	0.81	0.78	0.78
5L4O	3CW6	2.89	10	0.78	0.75	0.75
3GCA	6VUH	2.03	11	0.30	0.39	0.26
6VUH	3GCA	2.03	11	0.53	0.49	0.51
3L0U	6Y3G	2.52	12	0.73	0.69	0.73
6Y3G	3L0U	2.52	12	0.82	0.80	0.82
3OWI	3OWZ	3.04	13	0.81	0.76	0.77
3OWW	3OWZ	3.07	13	0.82	0.77	0.78
3OWZ	3OWI	3.04	13	0.84	0.77	0.81
3OWZ	3OWW	3.07	13	0.84	0.77	0.82
3OWZ	3OX0	3.02	13	0.83	0.76	0.81
3OWZ	3OXB	3.06	13	0.83	0.76	0.81
3OWZ	3OXD	3.03	13	0.83	0.76	0.80
3OWZ	3OXE	3.01	13	0.84	0.77	0.81
3OWZ	3OXJ	3.01	13	0.84	0.77	0.81
3OWZ	3OXM	2.96	13	0.83	0.76	0.81
3OX0	3OWZ	3.02	13	0.81	0.75	0.77
3OXB	3OWZ	3.06	13	0.81	0.75	0.77
3OXD	3OWZ	3.03	13	0.81	0.75	0.77
3OXE	3OWZ	3.01	13	0.81	0.76	0.77
3OXJ	3OWZ	3.01	13	0.81	0.76	0.77
3OXM	3OWZ	2.96	13	0.81	0.75	0.77
3TD0	3TD1	3.50	14	0.65	0.57	0.59
3TD1	3TD0	3.50	14	0.73	0.64	0.66
3WRU	4PDQ	3.34	15	0.77	0.77	0.78

Table K (continued)						
Input PDB code	Target PDB code	RMSD	Cluster index	ENCoM	Cut-ANM	PD-ANM
3WRU	6JBG	2.11	15	0.79	0.78	0.78
4GPY	4PDQ	3.78	15	0.70	0.68	0.69
4GPY	6JBG	2.77	15	0.75	0.77	0.76
4PDQ	3WRU	3.34	15	0.76	0.70	0.71
4PDQ	4GPY	3.78	15	0.71	0.62	0.64
4PDQ	6JBG	3.79	15	0.70	0.66	0.68
6JBG	3WRU	2.11	15	0.79	0.75	0.78
6JBG	4GPY	2.77	15	0.75	0.69	0.71
6JBG	4PDQ	3.79	15	0.73	0.69	0.71
4K31	4K32	3.18	16	0.75	0.73	0.74
4K32	4K31	3.18	16	0.75	0.73	0.75
4L81	4OQU	2.22	17	0.84	0.80	0.81
4OQU	4L81	2.22	17	0.85	0.78	0.79
4MSB	6Z18	2.24	18	0.95	0.55	0.61
4MSR	6WY3	2.10	18	0.90	0.53	0.61
4MSR	6Z18	2.44	18	0.94	0.53	0.67
5TDK	6Z18	2.38	18	0.92	0.50	0.71
6WY3	4MSR	2.10	18	0.91	0.87	0.89
6Z18	4MSB	2.24	18	0.95	0.94	0.94
6Z18	4MSR	2.44	18	0.94	0.89	0.93
6Z18	5TDK	2.38	18	0.91	0.89	0.91
4P3S	4P3T	3.40	19	0.68	0.63	0.64
4P3T	4P3S	3.40	19	0.68	0.60	0.66
4P3U	4P43	2.70	20	0.86	0.83	0.83
4P43	4P3U	2.70	20	0.89	0.86	0.90
4RZD	6XKN	2.13	21	0.81	0.71	0.71
6XKN	4RZD	2.13	21	0.81	0.70	0.71
6XKN	6XKO	2.07	21	0.82	0.72	0.72
6XKO	6XKN	2.07	21	0.80	0.72	0.72
4TZX	5E54	3.68	22	0.71	0.56	0.56
4TZX	5SWD	3.70	22	0.71	0.55	0.56
4TZY	5E54	3.74	22	0.71	0.58	0.58
4TZY	5SWD	3.75	22	0.72	0.57	0.58
4XNR	5E54	3.73	22	0.73	0.57	0.57
4XNR	5SWD	3.74	22	0.73	0.56	0.57
5E54	4TZX	3.68	22	0.57	0.49	0.49
5E54	4TZY	3.74	22	0.59	0.51	0.51
5E54	4XNR	3.73	22	0.42	0.36	0.36
5E54	5SWE	3.75	22	0.58	0.50	0.50
5E54	5UZA	3.78	22	0.59	0.52	0.52
5E54	6VWT	3.78	22	0.49	0.43	0.43
5E54	6VWV	4.02	22	0.61	0.55	0.55
5SWD	4TZX	3.70	22	0.57	0.49	0.49
5SWD	4TZY	3.75	22	0.58	0.50	0.50
5SWD	4XNR	3.74	22	0.42	0.36	0.36
5SWD	5SWE	3.76	22	0.58	0.50	0.50
5SWD	5UZA	3.78	22	0.59	0.51	0.51
5SWD	6VWT	3.79	22	0.49	0.43	0.43
5SWD	6VWV	4.04	22	0.61	0.55	0.55
5SWE	5E54	3.75	22	0.75	0.58	0.58

Table K (continued)						
Input PDB code	Target PDB code	RMSD	Cluster index	ENCoM	Cut-ANM	PD-ANM
5SWE	5SWD	3.76	22	0.75	0.58	0.58
5UZA	5E54	3.78	22	0.73	0.58	0.59
5UZA	5SWD	3.78	22	0.73	0.58	0.58
6VWT	5E54	3.78	22	0.69	0.58	0.58
6VWT	5SWD	3.79	22	0.69	0.58	0.58
6VWV	5E54	4.02	22	0.75	0.65	0.65
6VWV	5SWD	4.04	22	0.75	0.66	0.65
4Y1J	6CB3	25.96	23	0.98	0.96	0.96
6CB3	4Y1J	25.96	23	0.98	0.90	0.93
5ZEG	5ZEI	3.10	24	0.76	0.73	0.76
5ZEG	5ZEJ	3.19	24	0.74	0.70	0.72
5ZEG	5ZEM	3.01	24	0.77	0.76	0.77
5ZEI	5ZEG	3.10	24	0.77	0.80	0.80
5ZEJ	5ZEG	3.19	24	0.76	0.76	0.76
5ZEM	5ZEG	3.01	24	0.72	0.76	0.75
6C8D	6CAB	11.27	25	0.56	0.54	0.53
6CAB	6C8D	11.27	25	0.42	0.38	0.37
6DLR	6DNR	2.62	26	0.84	0.80	0.81
6DLS	6DNR	2.48	26	0.85	0.83	0.84
6DNR	6DLR	2.62	26	0.85	0.82	0.84
6DNR	6DLS	2.48	26	0.87	0.85	0.86
6E80	6E81	2.88	27	0.76	0.72	0.74
6E80	6E82	2.91	27	0.71	0.65	0.70
6E80	6E84	2.97	27	0.81	0.76	0.78
6E81	6E80	2.88	27	0.70	0.66	0.67
6E82	6E80	2.91	27	0.68	0.66	0.67
6E84	6E80	2.97	27	0.73	0.68	0.71
6N5K	6N5O	2.25	28	0.50	0.49	0.49
6N5O	6N5K	2.25	28	0.45	0.48	0.48
7EOI	7EOK	2.01	29	0.78	0.76	0.77
7EOK	7EOI	2.01	29	0.72	0.70	0.71

Table L: Performance on individual NMR ensembles for the ensemble variance benchmark.

PDB code	Cluster index	ENCoM NCO	Cut-ANM NCO	PD-ANM NCO	ENCoM RMSIP	Cut-ANM RMSIP	PD-ANM RMSIP
2LUB	1	0.83	0.81	0.82	0.74	0.72	0.73
1F5G	2	0.83	0.83	0.85	0.61	0.62	0.65
1F5H	2	0.78	0.78	0.77	0.62	0.58	0.58
2KYD	3	0.93	0.94	0.94	0.74	0.76	0.75
1AFX	4	0.60	0.56	0.58	0.48	0.47	0.47
6U79	5	0.65	0.67	0.70	0.60	0.61	0.64
2M21	6	0.78	0.71	0.73	0.62	0.58	0.59
1QET	7	0.75	0.88	0.80	0.61	0.69	0.69
2N7M	8	0.87	0.85	0.86	0.76	0.74	0.75
5KQE	9	0.57	0.51	0.51	0.59	0.53	0.55
2LUN	10	0.92	0.91	0.91	0.85	0.85	0.84
6VZC	10	0.94	0.92	0.92	0.81	0.79	0.79
2FDT	11	0.68	0.68	0.72	0.63	0.62	0.66
1JU1	12	0.95	0.91	0.92	0.82	0.76	0.77

Table L (continued)							
PDB code	Cluster index	ENCoM NCO	Cut-ANM NCO	PD-ANM NCO	ENCoM RMSIP	Cut-ANM RMSIP	PD-ANM RMSIP
6PK9	13	0.54	0.53	0.53	0.60	0.59	0.62
1K6G	14	0.75	0.74	0.79	0.57	0.58	0.61
1B36	15	0.94	0.91	0.92	0.84	0.78	0.79
28SP	16	0.75	0.75	0.75	0.71	0.72	0.72
1OW9	17	0.60	0.52	0.52	0.61	0.49	0.49
2JXQ	18	0.57	0.45	0.49	0.49	0.46	0.48
2JXS	18	0.83	0.87	0.85	0.63	0.64	0.64
6K84	19	0.32	0.36	0.37	0.37	0.41	0.40
2ADT	20	0.90	0.88	0.88	0.75	0.70	0.71
2JYF	20	0.90	0.84	0.86	0.80	0.73	0.74
2JYH	20	0.65	0.44	0.48	0.75	0.55	0.58
2JYJ	20	0.95	0.92	0.92	0.86	0.79	0.80
2RN1	21	0.75	0.70	0.71	0.69	0.67	0.70
1K4A	22	0.80	0.80	0.80	0.55	0.53	0.54
6XWJ	23	0.96	0.96	0.96	0.77	0.77	0.77
6XXA	23	0.86	0.86	0.86	0.69	0.69	0.70
3PHP	24	0.92	0.89	0.89	0.72	0.70	0.72
2MI0	25	0.91	0.87	0.88	0.69	0.61	0.63
1TBK	26	0.84	0.85	0.85	0.65	0.63	0.64
1YN1	26	0.83	0.81	0.83	0.64	0.59	0.62
6N8F	27	0.94	0.94	0.94	0.73	0.71	0.71
1GUC	28	0.70	0.65	0.65	0.57	0.57	0.57
1BN0	29	0.81	0.80	0.81	0.62	0.63	0.63
1LMV	30	0.92	0.92	0.92	0.73	0.71	0.69
1FHK	31	0.69	0.65	0.59	0.57	0.55	0.52
2NBZ	32	0.95	0.94	0.94	0.89	0.86	0.86
2GIO	33	0.84	0.81	0.81	0.72	0.67	0.67
2GIP	33	0.93	0.93	0.93	0.71	0.70	0.70
1SLP	34	0.83	0.75	0.81	0.59	0.56	0.60
1A9L	35	0.90	0.87	0.88	0.78	0.74	0.75
1U3K	35	0.92	0.90	0.90	0.79	0.74	0.74
2LX1	36	0.82	0.78	0.83	0.67	0.63	0.66
2KVN	37	0.80	0.79	0.79	0.53	0.52	0.51
6BG9	38	0.99	0.95	0.95	0.99	0.95	0.95
2Y95	39	0.75	0.71	0.72	0.64	0.59	0.61
2LP9	40	0.64	0.56	0.59	0.61	0.53	0.54
2LPA	40	0.71	0.71	0.72	0.60	0.63	0.63
1HLX	41	0.90	0.83	0.82	0.65	0.58	0.58
1E4P	42	0.94	0.92	0.93	0.74	0.69	0.70
2K3Z	43	0.58	0.55	0.57	0.53	0.51	0.52
2MXJ	44	0.68	0.71	0.72	0.52	0.54	0.54
1EBQ	45	0.80	0.69	0.75	0.75	0.64	0.72
1EBR	45	0.84	0.78	0.80	0.75	0.66	0.68
1EBS	45	0.84	0.76	0.79	0.79	0.67	0.70
5V17	46	0.64	0.58	0.59	0.67	0.61	0.61
6XWW	47	0.72	0.71	0.70	0.71	0.67	0.68
6XXB	47	0.70	0.69	0.69	0.64	0.62	0.63
1R2P	48	0.95	0.91	0.92	0.89	0.84	0.85
2LPS	48	0.88	0.86	0.87	0.83	0.82	0.83

Table L (continued)							
PDB code	Cluster index	ENCoM NCO	Cut-ANM NCO	PD-ANM NCO	ENCoM RMSIP	Cut-ANM RMSIP	PD-ANM RMSIP
1N66	49	0.87	0.82	0.86	0.67	0.64	0.67
2GRW	49	0.72	0.71	0.71	0.66	0.64	0.64
1ATW	50	0.53	0.46	0.55	0.59	0.47	0.55
2L5Z	51	0.75	0.69	0.71	0.63	0.59	0.59
1JU7	52	0.71	0.70	0.72	0.60	0.60	0.61
2M23	53	0.96	0.95	0.96	0.83	0.82	0.82
2L8F	54	0.87	0.85	0.85	0.76	0.72	0.73
1IDV	55	0.55	0.30	0.37	0.47	0.41	0.40
2LBL	56	0.65	0.65	0.69	0.58	0.63	0.65
2N3Q	57	0.96	0.94	0.95	0.86	0.82	0.84
2M24	58	0.77	0.70	0.74	0.61	0.54	0.58
2IXY	59	0.75	0.70	0.70	0.62	0.58	0.57
2N6X	60	0.96	0.94	0.95	0.91	0.84	0.90
2IRO	61	0.56	0.54	0.54	0.52	0.50	0.51
1Z31	62	0.66	0.56	0.57	0.64	0.56	0.57
2NC1	63	0.96	0.93	0.94	0.92	0.89	0.89
4A4S	64	0.90	0.86	0.87	0.82	0.77	0.77
4A4T	64	0.88	0.86	0.86	0.79	0.76	0.77
4A4U	64	0.93	0.93	0.92	0.81	0.79	0.79
2N4L	65	0.69	0.66	0.67	0.63	0.60	0.61
2F88	66	0.78	0.76	0.77	0.75	0.73	0.74
2LPT	66	0.91	0.89	0.90	0.80	0.79	0.79
2LC8	67	0.74	0.60	0.61	0.65	0.56	0.57
2NCI	68	0.77	0.70	0.72	0.72	0.64	0.65
1YLG	69	0.63	0.56	0.56	0.58	0.54	0.54
1YNC	69	0.71	0.66	0.67	0.58	0.54	0.56
1YNE	69	0.67	0.64	0.66	0.56	0.51	0.53
1YNG	69	0.59	0.58	0.58	0.55	0.55	0.56
1TJZ	70	0.91	0.88	0.90	0.63	0.61	0.63
1T4X	71	0.64	0.38	0.40	0.66	0.47	0.49
1MFJ	72	0.76	0.78	0.78	0.65	0.66	0.67
2D18	73	0.90	0.92	0.93	0.72	0.75	0.78
2D19	73	0.86	0.83	0.84	0.73	0.68	0.69
2RLU	74	0.56	0.55	0.58	0.57	0.56	0.57
1F7F	75	0.90	0.86	0.87	0.78	0.72	0.73
1F7G	75	0.88	0.81	0.83	0.71	0.62	0.64
6HYK	76	0.78	0.81	0.87	0.78	0.76	0.78
2AHT	77	0.94	0.77	0.86	0.76	0.63	0.68
2M22	78	0.76	0.75	0.76	0.60	0.58	0.60
1RNG	79	0.53	0.53	0.51	0.52	0.51	0.51
1ATO	80	0.65	0.66	0.65	0.53	0.53	0.53
2KHY	81	0.93	0.91	0.91	0.76	0.72	0.72
1ZIF	82	0.56	0.55	0.57	0.53	0.50	0.51
1WKS	83	0.61	0.67	0.68	0.45	0.51	0.53
1XHP	84	0.78	0.74	0.74	0.70	0.64	0.64
2O33	85	0.69	0.70	0.70	0.56	0.56	0.55
1A60	86	0.89	0.87	0.89	0.72	0.68	0.70
2GM0	87	0.91	0.89	0.89	0.84	0.81	0.82
1Q75	88	0.60	0.70	0.70	0.50	0.52	0.53

Table L (continued)							
PDB code	Cluster index	ENCoM NCO	Cut-ANM NCO	PD-ANM NCO	ENCoM RMSIP	Cut-ANM RMSIP	PD-ANM RMSIP
2LV0	89	0.93	0.93	0.92	0.77	0.79	0.78
1F85	90	0.85	0.85	0.85	0.61	0.61	0.59
5LSN	91	0.91	0.91	0.91	0.90	0.90	0.90
2M4W	92	0.62	0.52	0.53	0.55	0.52	0.52
1ZIG	93	0.71	0.71	0.72	0.58	0.58	0.60
2N6S	94	0.91	0.87	0.86	0.83	0.81	0.80
6GE1	95	0.57	0.61	0.57	0.53	0.53	0.52
5A17	96	0.60	0.58	0.59	0.58	0.56	0.58
5A18	96	0.62	0.60	0.64	0.64	0.62	0.66
1LDZ	97	0.90	0.84	0.87	0.65	0.59	0.61
2KY1	98	0.81	0.79	0.78	0.77	0.74	0.73
1IE1	99	0.55	0.52	0.52	0.48	0.43	0.43
1IE2	99	0.64	0.64	0.65	0.51	0.50	0.51
2MXL	100	0.85	0.84	0.84	0.71	0.70	0.70
2FEY	101	0.75	0.69	0.71	0.66	0.63	0.64
1F84	102	0.84	0.85	0.86	0.65	0.64	0.66
1QES	103	0.88	0.86	0.86	0.69	0.69	0.69
2G1W	104	0.66	0.64	0.66	0.55	0.56	0.56
1TXS	105	0.76	0.70	0.72	0.64	0.59	0.61
1JO7	106	0.90	0.84	0.86	0.69	0.60	0.63
1MFY	106	0.88	0.82	0.83	0.75	0.66	0.68
5VH7	107	0.93	0.92	0.92	0.74	0.74	0.73
2KOC	108	0.68	0.71	0.71	0.54	0.55	0.54
6BY4	108	0.63	0.74	0.69	0.53	0.61	0.57
6BY5	108	0.55	0.61	0.59	0.51	0.55	0.52
5V2R	109	0.93	0.91	0.91	0.68	0.63	0.64
2EUY	110	0.92	0.91	0.92	0.75	0.72	0.72
1AQO	111	0.83	0.75	0.77	0.65	0.57	0.59
1NBR	111	0.72	0.68	0.68	0.64	0.59	0.60
1VOP	112	0.31	0.42	0.49	0.41	0.49	0.55
1QWA	113	0.86	0.83	0.83	0.67	0.66	0.66
2HNS	114	0.92	0.89	0.91	0.70	0.66	0.68
1A3M	115	0.79	0.81	0.81	0.69	0.73	0.74
2NBX	116	0.92	0.89	0.89	0.89	0.85	0.86
5N5C	117	0.77	0.74	0.75	0.50	0.48	0.48
1R7W	118	0.82	0.76	0.77	0.65	0.57	0.58
1R7Z	118	0.89	0.85	0.85	0.76	0.67	0.69
2NC0	119	0.93	0.91	0.92	0.79	0.75	0.76
6N8I	120	0.93	0.91	0.91	0.74	0.71	0.71
2KPC	121	0.76	0.73	0.73	0.55	0.51	0.53
2JXV	122	0.88	0.89	0.89	0.80	0.80	0.81
2KRQ	123	0.73	0.65	0.67	0.63	0.58	0.61
1M82	124	0.86	0.85	0.85	0.60	0.60	0.60
2MFD	125	0.64	0.65	0.65	0.53	0.54	0.53
1P5O	126	0.93	0.90	0.91	0.85	0.80	0.82
5KMZ	127	0.60	0.60	0.61	0.58	0.56	0.58
2M18	128	0.53	0.55	0.57	0.49	0.55	0.56
6AAS	129	0.64	0.57	0.65	0.46	0.49	0.51
2L1F	130	0.62	0.60	0.60	0.58	0.57	0.58

Table L (continued)							
PDB code	Cluster index	ENCoM NCO	Cut-ANM NCO	PD-ANM NCO	ENCoM RMSIP	Cut-ANM RMSIP	PD-ANM RMSIP
2RQJ	131	0.39	0.34	0.35	0.45	0.41	0.40
2PCV	132	0.93	0.91	0.91	0.82	0.80	0.80
2LK3	133	0.81	0.73	0.83	0.75	0.72	0.76
1YSV	134	0.70	0.65	0.69	0.62	0.56	0.59
2MXK	135	0.75	0.75	0.74	0.59	0.60	0.60
1MT4	136	0.83	0.80	0.81	0.61	0.58	0.61
2LJJ	137	0.65	0.63	0.64	0.61	0.58	0.59
1P5M	138	0.93	0.88	0.89	0.81	0.74	0.75
2RVO	139	0.65	0.61	0.63	0.60	0.57	0.60
2IXZ	140	0.39	0.47	0.52	0.38	0.47	0.47
1LC6	141	0.75	0.74	0.75	0.59	0.58	0.59
1NC0	141	0.74	0.70	0.71	0.62	0.58	0.58
1SY4	141	0.86	0.85	0.85	0.69	0.66	0.66
1SYZ	141	0.63	0.65	0.66	0.62	0.59	0.60
2KEZ	141	0.73	0.68	0.69	0.66	0.61	0.61
2KF0	141	0.77	0.70	0.74	0.76	0.71	0.72
1M5L	142	0.95	0.89	0.89	0.83	0.77	0.77
1YMO	143	0.79	0.75	0.77	0.65	0.60	0.62
2K95	143	0.83	0.80	0.80	0.66	0.63	0.63
2K96	143	0.76	0.75	0.75	0.64	0.63	0.63
7DD4	144	0.74	0.72	0.75	0.70	0.69	0.72
2MNC	145	0.84	0.81	0.82	0.57	0.51	0.54
2LQZ	146	0.69	0.61	0.65	0.61	0.55	0.58
1S34	147	0.44	0.35	0.35	0.49	0.49	0.49
1N8X	148	0.85	0.84	0.85	0.74	0.71	0.73
2KUW	149	0.95	0.90	0.90	0.87	0.79	0.80
2K5Z	150	0.74	0.76	0.77	0.69	0.70	0.71
2OJ7	151	0.24	0.45	0.42	0.37	0.40	0.39
2OJ8	151	0.42	0.49	0.53	0.40	0.48	0.49
2LDL	152	0.84	0.72	0.77	0.74	0.65	0.69
2MTJ	153	0.97	0.94	0.95	0.91	0.85	0.86
5IEM	154	0.89	0.85	0.87	0.82	0.78	0.80
1P5N	155	0.77	0.74	0.77	0.64	0.61	0.64
5VH8	156	0.91	0.90	0.90	0.71	0.72	0.71
2D17	157	0.81	0.74	0.76	0.69	0.63	0.65
2JSE	158	0.70	0.71	0.76	0.67	0.68	0.71
2KZL	159	0.89	0.87	0.87	0.82	0.81	0.80
1CQL	160	0.92	0.88	0.89	0.80	0.76	0.77
6VAR	161	0.87	0.86	0.87	0.78	0.77	0.78
2PCW	162	0.90	0.89	0.86	0.74	0.72	0.68
2KUU	163	0.95	0.93	0.94	0.89	0.85	0.85
6W3M	164	0.72	0.71	0.72	0.70	0.68	0.71
1OSW	165	0.69	0.69	0.68	0.56	0.54	0.53
1SCL	166	0.93	0.88	0.87	0.83	0.80	0.79
2IRN	167	0.60	0.62	0.59	0.56	0.59	0.59
2KE6	168	0.96	0.95	0.95	0.90	0.88	0.88
2KUR	168	0.94	0.93	0.93	0.84	0.83	0.83
1Z2J	169	0.94	0.93	0.93	0.81	0.78	0.79
2GV4	170	0.82	0.70	0.81	0.72	0.68	0.73

Table L (continued)							
PDB code	Cluster index	ENCoM NCO	Cut-ANM NCO	PD-ANM NCO	ENCoM RMSIP	Cut-ANM RMSIP	PD-ANM RMSIP
1K4B	171	0.45	0.41	0.44	0.54	0.50	0.52
1BVJ	172	0.70	0.62	0.68	0.62	0.57	0.63
2KD8	173	0.78	0.74	0.75	0.66	0.62	0.64
2P89	174	0.91	0.87	0.89	0.77	0.71	0.73
1K6H	175	0.79	0.79	0.79	0.58	0.61	0.60
2KRP	176	0.86	0.68	0.75	0.65	0.57	0.58
6VA1	177	0.83	0.84	0.82	0.62	0.62	0.61
2JTP	178	0.88	0.87	0.88	0.66	0.62	0.64
2N8V	179	0.64	0.61	0.64	0.60	0.59	0.63
2MQT	180	0.87	0.86	0.87	0.80	0.78	0.79
1QWB	181	0.71	0.63	0.64	0.61	0.54	0.55
5UZT	182	0.87	0.79	0.80	0.75	0.65	0.68
2L3E	183	0.72	0.67	0.68	0.63	0.58	0.59
1ATV	184	0.28	0.25	0.24	0.47	0.40	0.40
1IKD	185	0.91	0.86	0.87	0.62	0.58	0.58
1I3X	186	0.62	0.47	0.59	0.54	0.46	0.52
2GVO	187	0.74	0.70	0.71	0.54	0.51	0.51
2JR4	188	0.64	0.61	0.59	0.55	0.50	0.49
1FYO	189	0.93	0.93	0.93	0.72	0.73	0.72
1TLR	190	0.69	0.65	0.67	0.58	0.53	0.57
2QH4	191	0.83	0.86	0.86	0.58	0.60	0.60
2LHP	192	0.86	0.85	0.86	0.72	0.72	0.73
2K41	193	0.49	0.46	0.47	0.51	0.47	0.47
1MFK	194	0.73	0.72	0.74	0.55	0.57	0.59
1KKA	195	0.77	0.73	0.72	0.66	0.63	0.63
2H49	196	0.59	0.57	0.59	0.53	0.52	0.54
7K4L	197	0.82	0.65	0.71	0.71	0.61	0.66
2MEQ	198	0.65	0.59	0.63	0.57	0.53	0.55
2KPD	199	0.70	0.68	0.69	0.57	0.54	0.55
5KH8	200	0.71	0.57	0.61	0.67	0.58	0.59
1OQ0	201	0.83	0.82	0.88	0.57	0.56	0.59
1NA2	202	0.92	0.92	0.92	0.77	0.74	0.74
2KXZ	203	0.96	0.96	0.96	0.90	0.90	0.90
5WQ1	204	0.45	0.47	0.50	0.42	0.42	0.45
1ANR	205	0.79	0.69	0.73	0.59	0.53	0.56
7JU1	205	0.76	0.67	0.69	0.56	0.49	0.50
2F87	206	0.54	0.55	0.54	0.49	0.51	0.51
2N6T	207	0.86	0.84	0.84	0.79	0.76	0.76
2F4X	208	0.84	0.90	0.91	0.64	0.66	0.66
1ELH	209	0.91	0.78	0.86	0.84	0.74	0.79
1JOX	210	0.75	0.66	0.69	0.56	0.55	0.54
1JP0	210	0.90	0.89	0.89	0.59	0.54	0.56
2JWV	211	0.97	0.97	0.96	0.85	0.83	0.83
2L2J	212	0.94	0.94	0.94	0.82	0.82	0.83
1RRR	213	0.65	0.70	0.71	0.55	0.59	0.61
2K66	214	0.95	0.79	0.81	0.80	0.70	0.69
2M12	215	0.57	0.51	0.53	0.54	0.46	0.48
2N7X	216	0.87	0.86	0.86	0.63	0.62	0.63
1ESY	217	0.59	0.60	0.63	0.50	0.52	0.54

Table L (continued)							
PDB code	Cluster index	ENCoM NCO	Cut-ANM NCO	PD-ANM NCO	ENCoM RMSIP	Cut-ANM RMSIP	PD-ANM RMSIP
1KKS	218	0.79	0.57	0.56	0.66	0.52	0.52
2LU0	219	0.89	0.85	0.86	0.78	0.73	0.75
2KPV	220	0.68	0.64	0.64	0.69	0.63	0.64
2K65	221	0.90	0.92	0.92	0.69	0.70	0.68
1QC8	222	0.72	0.64	0.66	0.51	0.44	0.46
5V16	223	0.77	0.73	0.72	0.81	0.78	0.79
2JYM	224	0.85	0.83	0.84	0.68	0.64	0.67
1K5I	225	0.83	0.82	0.83	0.72	0.70	0.71
2LAC	226	0.86	0.82	0.86	0.70	0.63	0.70
1A51	227	0.97	0.94	0.94	0.90	0.84	0.86
1S9S	228	0.94	0.90	0.91	0.89	0.85	0.86
1BGZ	229	0.69	0.80	0.79	0.66	0.70	0.71
2XEB	230	0.52	0.41	0.44	0.66	0.60	0.63
2KBP	231	0.44	0.51	0.51	0.43	0.45	0.46
1ROQ	232	0.85	0.88	0.88	0.65	0.61	0.61
2N6W	233	0.94	0.93	0.93	0.86	0.82	0.83
2LBK	234	0.66	0.65	0.68	0.59	0.61	0.61
2GV3	235	0.74	0.60	0.65	0.61	0.54	0.55
1ZC5	236	0.94	0.91	0.91	0.79	0.78	0.78
1MNX	237	0.93	0.91	0.91	0.81	0.80	0.79
1JTJ	238	0.91	0.87	0.88	0.64	0.56	0.59
1JUR	239	0.70	0.65	0.66	0.65	0.63	0.63
1Z30	240	0.85	0.83	0.82	0.66	0.62	0.63
2M8K	241	0.77	0.73	0.75	0.63	0.57	0.60
2M57	242	0.87	0.84	0.86	0.69	0.63	0.66
17RA	243	0.97	0.96	0.96	0.82	0.80	0.79
2N2P	244	0.74	0.54	0.60	0.64	0.51	0.54
2B7G	245	0.65	0.60	0.64	0.66	0.61	0.63
1HWQ	246	0.87	0.85	0.86	0.68	0.65	0.66
1ZIH	247	0.64	0.62	0.63	0.56	0.53	0.53
1UUU	248	0.69	0.58	0.63	0.56	0.50	0.55
1E95	249	0.89	0.82	0.85	0.71	0.64	0.66
2KRL	250	0.70	0.64	0.65	0.69	0.63	0.64
2HEM	251	0.87	0.79	0.81	0.74	0.70	0.72
2LDT	252	0.81	0.78	0.80	0.68	0.63	0.64
2QH3	253	0.82	0.82	0.83	0.61	0.57	0.58
2RPT	254	0.94	0.92	0.92	0.75	0.71	0.71
6MXQ	255	0.88	0.86	0.86	0.82	0.81	0.81
5UF3	256	0.68	0.70	0.73	0.76	0.78	0.80
2L6I	257	0.53	0.48	0.49	0.54	0.52	0.51
2HUA	258	0.93	0.90	0.90	0.87	0.83	0.83
1D0U	259	0.87	0.72	0.74	0.62	0.56	0.57
2M5U	260	0.96	0.96	0.96	0.83	0.80	0.79
2EVY	261	0.60	0.56	0.55	0.61	0.60	0.60
2QH2	262	0.88	0.83	0.85	0.65	0.61	0.62
2KY2	263	0.91	0.90	0.90	0.71	0.70	0.70
2N2O	264	0.67	0.47	0.51	0.64	0.46	0.50
7LVA	265	0.93	0.92	0.92	0.90	0.88	0.89
6NOA	266	0.83	0.82	0.83	0.76	0.74	0.76

Table L (continued)							
PDB code	Cluster index	ENCoM NCO	Cut-ANM NCO	PD-ANM NCO	ENCoM RMSIP	Cut-ANM RMSIP	PD-ANM RMSIP
2LBJ	267	0.64	0.55	0.56	0.67	0.56	0.56
1PJY	268	0.87	0.86	0.85	0.63	0.60	0.59
2ES5	269	0.81	0.62	0.66	0.64	0.52	0.54
2KY0	270	0.87	0.86	0.85	0.81	0.76	0.75
2NBY	271	0.97	0.94	0.96	0.86	0.84	0.85

Table M: MD trajectories of miR-125a variants.

Base pair 22	Replicate	Simulation time (ns)
AA	1	122.6
AA	2	122.4
AA	3	120.3
AC	1	123.6
AC	2	135.4
AC	3	123.2
AG	1	107.4
AG	2	110.4
AG	3	121.2
AU	1	123.4
AU	2	123.2
AU	3	110.2
CA	1	123.2
CA	2	122.4
CA	3	123.3
CC	1	110.5
CC	2	111.1
CC	3	112.1
CG	1	135.4
CG	2	124.1
CG	3	123.2
CU	1	125.3
CU	2	111.4
CU	3	123.1
GA	1	109.5
GA	2	112.2
GA	3	109.1
GC	1	124.5
GC	2	124.1
GC	3	112.5
GG	1	112.4
GG	2	108.8
GG	3	137.4
GU	1	123.4
GU	2	111.5
GU	3	112.1
UA	1	112.4
UA	2	122.3
UA	3	122.2

Table M (continued)		
Base pair 22	Replicate	Simulation time (ns)
UC	1	132.1
UC	2	134.1
UC	3	119.1
UG	1	112.1
UG	2	123.5
UG	3	124.3
UU	1	124.1
UU	2	121.2
UU	3	121.6

Table N: ENCoM interaction strength for different base pairs. The base pairs are taken from the WT miR-125a structure.

Base pair	Interaction strength
AC	134.9
AG	0.0
AU	167.8
CG	218.0
CU	150.7
GU	149.4
UU	53.7

REFERENCES

[1] Ranhui Duan, ChangHui Pak, and Peng Jin. Single nucleotide polymorphism associated with mature miR-125a alters the processing of pri-miRNA. *Human Molecular Genetics*, 16(9):1124–1131, mar 2007.

[2] Alan A Chen and Angel E García. High-resolution reversible folding of hyperstable rna tetraloops using molecular dynamics simulations. *Proceedings of the National Academy of Sciences*, 110(42):16820–16825, 2013.

[3] Mark James Abraham, Teemu Murtola, Roland Schulz, Szilárd Páll, Jeremy C Smith, Berk Hess, and Erik Lindahl. Gromacs: High performance molecular simulations through multi-level parallelism from laptops to supercomputers. *SoftwareX*, 1:19–25, 2015.

[4] Magdalena Rother, Kristian Rother, Tomasz Puton, and Janusz M. Bujnicki. ModeRNA: a tool for comparative modeling of RNA 3d structure. *Nucleic Acids Research*, 39(10):4007–4022, feb 2011.

[5] Paul Dallaire, Huiping Tan, Keith Szulwach, Christopher Ma, Peng Jin, and François Major. Structural dynamics control the MicroRNA maturation pathway. *Nucleic Acids Res*, page gkw793, sep 2016.

2 LIST OF LEGENDS

Fig. A: Correlation between miR-125a maturation efficiency and Dynamical Signature distance to WT for both ENCoM and MD simulations. The average distance for all 9 possibilities is reported for the MD simulations (3 replicates for the variant, 3 replicates for

WT). The mean-square fluctuation was computed at P, C1′ and C2 atoms as the square of the RMSF computed with GROMACS. The ENCoM Entropic Signature was computed at $\beta = e^{0.25}$, the value selected from the B-factors benchmark. The Pearson linear correlation coefficients reported do not reach statistical significance.

Fig. B: Cut-ANM and PD-ANM parameter sweep for the B-factors benchmark. Parameters are interaction distance cutoff (in Å) for Cut-ANM and power dependence for PD-ANM. Both are varied in increments of 0.5 from their minimum practical value up to 20.

Fig. C: Performance of multiple linear regression on an 80-20 train-test split. A) Predictive R2 for each model alone or in combination with the MC-Fold enthalpy of folding. For the three ENMs, scaling factors for the dynamical signature were explored around the value which gave the best respective performance in the B-factors benchmark. Predictive R2 values below 0 are shown in gray. B) Performance on the test set for the best combination of parameters for every model alone or in tandem with MC-Fold.

Fig. D: Size distributions of the sequence clusters from the 3 benchmarks. A) B-factors benchmark. B) Conformational change from X-ray structures benchmark. C) NMR ensemble structural variance benchmark.

Fig. E: Number of nontrivial normal modes at 5% for the conformational change (overlap) and NMR ensemble variance benchmarks.

Fig. F: Simulation of predictive R-squared from combinations of MC-Fold enthalpy with random noise. Each of the 10 000 iterations represents the best predictive R2 value from 656 samplings of 116 values from a gaussian distribution centered around the training set mean and with a standard deviation two times smaller. The noise is then averaged at each sampling with the MC-Fold predictions and predictive R2 is computed. The red line shows the R2 of the ENCoM-MC-Fold combination on the hard benchmark (0.215).

Fig. G: Computational cost of the ENMs on the Thermus thermophilus 30S ribosomal subunit and on miR-125a. A) Thermus thermophilus 30S ribosomal subunit. B) MiR-125a.

Table H: Atom type assignation of the four standard nucleotides. The atom types assigned are the ones from Sobolev and coworkers, divided in eight classes: hydrophilic, acceptor, donor, hydrophobic, aromatic, neutral, neutral-donor and neutral-acceptor.

Table I: PDB codes of the structures used for the three benchmarks

Table J: Performance on individual structures for the B-factors correlation benchmark

Table K: Performance on individual pairs of conformations for the X-ray conformational change benchmark.

Table L: Performance on individual NMR ensembles for the ensemble variance benchmark.

Table M: MD trajectories of miR-125a variants.

Table N: ENCoM interaction strength for different base pairs. The base pairs are taken from the WT miR-125a structure.
