# Supplementary material for: Sequence-sensitive elastic network captures dynamical features necessary for miR-125a maturation
Source: PLoS Comput Biol. 2022 Dec 14;18(12):e1010777. doi: 10.1371/journal.pcbi.1010777 (PMC9797095; doi:10.1371/journal.pcbi.1010777)
Supplement: Attachment — Submitted filename: Answer_Reviewers_RJN_OM.pdf [file pcbi.1010777.s006.pdf]

Université de Montréal Department of Pharmacology and Physiology **Rafael Najmanovich, PhD**

Pavillon Paul-G.-Desmarais
2960 Chemin de la Tour
Montreal, H3T 1J4 QC -
Canada
(514) 343-6345 or (514)-582-7552
rafael.najmanovich@umontreal.ca
https://nrglab.github.io
@RNajmanovich

October 28, 2022

Dr. Shi-Jie Chen
Academic Editor
PLOS computational Biology

Dr. Arne Elofsson
Section Editor
PLOS computational Biology

Re: Submission of a revised version of manuscript PCOMPBIOL-D-22-00886

Dear Dr. Shi-Jie Chen and Dr. Arne Elofsson

First, we would like to thank the editors and the three reviewers for their thorough reading of our manuscript and numerous insightful comments. In addition, we are grateful that they wished to remain part of the review process despite the granted extension. We believe that as a result of addressing their comments, we have made our work much clearer by implementing the reviewer suggestions.

In what follows we respond individually to each concern expressed by the editor and each of the three reviewers. We believe that in its current, improved form, the manuscript will be found to be acceptable for publication.

M[illegible] [illegible] [illegible] [illegible] E[illegible] [illegible] R[illegible] #[illegible]

In particular, we do think a comparison with MD-simulations is necessary (see reviewer comments)

The main concern which emerged from the review was the needed comparison with molecular dynamics simulations. We already had simulated the dynamics of 16 miR-125a variants extensively but had not included the data in the original manuscript. The simulations were analyzed and the supplementary material now contains the detailed methodology for these simulations as well as a comparison with ENCoM. As described in the supplementary data, we simulated nearly 5.75 µs in total for the 16 possible miR-125a variants at base pair 22 in triplicate. This is the position of the G22U mutation that leads to poor prognoosis in breast cancer. As reported in the supplementary data, we calculated the correlations between dynamical

signatures (local fluctuations) differences between variants and wildtype with respect to maturation efficiences. The more different the dynamics, the poorer the maturation efficiency is expected to be, leading to an expected anticorrelation between dynamical signature or MD mean square fluctuations and experimental values. While there is not enough data with 16 points to obtain statistically significant results, the correlations obtained are poored for MD simulations (R=0.05) than that obtained using ENCoM Entropic Signature (R=-0.27).

Given the poor results obtained with MD, we believe that they should be presented in the supplementary data instead of the main text and only metion the high computational cost (40 core-years) and nonsignificant performance (as ENCoM) for this small subset of substitutions. Even if MD could offer a sizeable advantage for maturation efficiency prediction (it may in principle do at long enough timescales), we want to emphasize the ultra-high-throughput applicability of our work. For instance, at approximately 3 seconds computational cost per variant, millions of theoretical mutations could be screened on a modestly sized compute cluster. While the comparison to MD is indeed needed and interesting, even enhanced sampling or coarse-grained MD cannot hope to achieve such high throughput.

R[illegible] [illegible] R[illegible] #[illegible]

The present work offers an interesting coarse grained ENM-based approach to approximate the local dynamics of RNAs. This approach is demonstrated to be sufficiently cheap for large-scale applications while able to predict sequence-specific properties. Using this approach, the authors sucessfully related the dynamics of pri-miR-125a variants to its maturation efficiency. Yet this exciting achievement appears buried in the details and not stressed enough. Moreover, to make this manuscript more convincing, extra evidences shall be provided.

We modified the text throughout to bring the message across more clearly and further added one extra benchmark of miR-125a maturation efficiency prediction, helping to show the advantage of using this approach, with high R^2 values obtained.

Although molecular dynamics simulations could be too expensive for large-scale validations, a comparison and this approach and MD simulations is still necessary, at least for the wild-type miR-125a and one/two variant sequences. This won't be super costly, since the system is not huge and only local dynamics around the medoid structure of pri-miR-125a need to be sampled. Such comparison not only adds more credit to ENCoM but may also offer clues on future improvements of including electro-static terms.

As suggested, we have added a comparison with MD simulations to the supplementary material. However, we do not think only local dynamics need to be sampled. In fact, we believe the reason for the poor performance of MD is precisely the fact that ENCoM samples much longer timescales/large conformational changes through the calculation of a dynamical signatures using normal modes,
whereas MD simulations, even the approximate 100 ns per variant (for 16 variants) do not sufficiently sample the conformational space of a primary microRNA transcript.

A related interesting question could be at what temperature shall the MD be performed and what is the relation between this temperature with the parameter beta and the entropy of the molecule.
Some discussion on this will be helpful.

We agree that it would have been insightful to explore many MD temperatures. However, the computational cost for this system is fairly high at around 8 core-years per µs simulation time. For this reason, we feel that such an analysis is outside the scope of the present manuscript, which focuses on a very fast method for computational dynamics applicable in a high-throughput fashion. *"The pinpointing of important regions in the pri-miR-125a" shall be stressed more. This could be done by (1) starting a new paragraph at Line 484 from "Strikingly, ..."; (2) Modify Figure 7 so that the GHG motif can stand out from the rest.*

We have implemented the suggested changes, both to the text and Figure 7.

Other than these, I find the manuscript clearly written and scientifically sound.

R[illegible] [illegible] R[illegible] #[illegible]

In their manuscript, Maillot et al introduce an RNA-version of an elastic network model (ENCoM) originally developed for proteins. They evaluate the accuracy of the model in predicting B-factors and motions as described by NMR bundles.

To clarify, in addition to B-factors and motions from NMR ensembles, we also use pairs of crystal structures (the "conformational change" benchmark).

Furthermore, they train a regularised linear model to predict maturation efficiency of a miRNA using as input features dynamical information predicted from the elastic network model.

That is correct. Additionally, the LASSO linear regression allows us to determine the contributions of unique positions in the dynamical signature.

While some of the aspects of this work are innovative, I have a number of concerns.

We appreciate the recognition of the innovative aspects of the work and address in detail the concerns below.

If I understand correctly, the ENCoM model is based on three beads: C1', C2, and P. What are the parameters of the model in eq. 1? While some of the scaling parameters may be adapted from proteins, there is no P in proteins. Also, how were the equilibrium distances/angles (e.g. r0, theta0, etc in eq 1) determined? Since C1'/C2/P are not covalently bonded there is no simple way to determine those parameters e.g. from known RNA structures.

We realized that eq. 1 was unclear, especially to readers more familiar with MD potentials. We extensively clarified the text around it. In essence, as a harmonic potential, the ENCoM potential just describes a 3N-dimensional (N being the number of beads) energy well around the input structure, which has zero energy. There are only four parameters in the model: a weight associated with each term. Their values are now given in the text and the number four is explicitly mentioned.

The equilibrium distances, angles, dihedrals etc. are simply those found in the input structure (minimum energy in the model). While the beads are not covalently bonded, neither are two consecutive alpha carbons when considering the ENCoM model for proteins. Yet there still exists a dihedral angle for 4 consecutive alpha carbons. The distance, angle and dihedral angle terms are not representing those created by consecutive atoms covalently bound but by consecutive beads. We have clarified the text around Eq. 1 to reflect this.

The model is based on a simple atom type system developed by Sobolev *et al.* (Proteins, 25(1):120–129, 1996.), as outlined in the original article ENCoM article (Frappier, V. and Najmanovich, R. PLoS Comput Biol, 10(4):e1003569, 2014). These atom types interact in a binary fashion, further simplifying the model. Thus, we have assigned the "neutral-acceptor" atom type to RNA phosphorous atoms. We have clarified the text around Eq. 2 to better explain the surface complementarity term. We added extensive discussion about both the coarse-grained nature of all ENMs, and the "geometric hypothesis" of their relatively high performance: that ultimately, the very shape of a biomolecule dictates a good portion of accessible motions. In that sense, ENCoM adds layers of relevant geometric information - the connectivity of the residues, and the interactions between residues sharing atomic contacts. We have also added an analysis of the potential's sequence sensitivity in Figure 1, which highlights that ENCoM does, despite the simple 8 atom type system, capture the higher rigidity of a GC base pair as opposed to an AU base pair. Supplementary Table 7 gives the interaction term for all base pairs in miR-125a, and again noncanonical base pairs are weaker than canonical ones. For now, we believe we have sufficiently demonstrated that while not perfect, ENCoM's sequence sensitivity does capture the most important features of RNA base pairing.

Additionally, the improvements with respect to simpler models with much fewer parameters is only marginal, it is thus not clear why one should use ENCoM rather than an ANM.

While it is true that improvements on the conformational change and NMR ensemble variance benchmarks conducted are marginal, the other tested models are unable to properly capture the effects of mutations as our work demonstrates. This is in line with the previous work on proteins, and there lies the advantage of our method. We think the title of the paper and the focus on miR-125a highlight this main takeaway from our work. Maybe reviewer #2 was under the impression that there are more parameters in ENCoM than what there really is (4). We believe clarifying Eq. 1 and 2 helped with this issue. To be clear, ENCoM's 4 parameters as opposed to 1 parameter for ANM, offer slight improvement on the prediction of conformational changes and ensemble variance but make it possible to predict the effect of mutations on function as a result of a more realistic representation of non-bonded interactions, as shown in Figure 1 C-D. We have clarified the discussion around the differences with respect to ANM and why we think ENCoM's advantages exist throughout the text.
Of relevance are Figure 1 C-D, Figure 4 C-D, and the first discussion section.

2. I like the idea of using the entropic signature as features in a LASSO model to predict miRNA maturation, but the authors should explain and motivate their choice more clearly. If I understand correctly, the underlying idea is that each miRNA sequence display a given internal dynamics which is (assumed to be) tightly linked to its maturation efficiency. Since the internal dynamics could be captured by the 'entropic signature', one should be able to predict maturation efficiency from ENCoM. While I understand and appreciate that the authors constructed a non-trivial test dataset, the resulting R2 is very low, suggesting that the model does not generalize to a sufficient degree. As such, I am not convinced that the ENCoM model can help the prediction of maturation efficiency. In order to improve the model, the authors can consider to introduce additional features to the model (e.g. the ensemble diversity as predicted from sequence, others?)

These are very valid points, and the reason for the addition of the inverted benchmark (Figure 7). ENCoM reahces 0.34 R2 on its own, and 0.66 with MC-Fold. This is more representative of the expected performance in a predictive application, where the model would be trained from all available data. We believe showing that ENCoM's performance edge extends to this more realistic benchmark clarifies the issue. Also, as stated in the new text surrounding that figure, while ANM does capture some signal, it arises from the

small differences in the cartesian coordinates of the nucleobase beads which uniquely identify each of the four nucleobases for every position. The fact that these merely represent some complex transformation of sequence information is apparent from the very slight gain over the sequence vector when combined with MC-Fold (3% vs 10% for ENCoM).

Minor points: - the NMR bundle does not directly represent dynamics, but rather a collection of structural models that all agree to a certain extent to the measured data. As such, NMR bundles do not necessarily represent the internal structural dynamics.

Point taken. We have modified the text to better reflect this fact.

- Check several typos throughout the manuscript (chages -> changes on page 3, line 80. Diplacement - -> displacement line 189 page 6. etropic -> entropic page 13 line 450, constructed -> constructed - page 9 , line 286)

The manuscript was checked again and the typos above were corrected.

R[illegible] [illegible] R[illegible] #[illegible]

In this paper, the authors used a variation of the Elastic Network Model that includes sequence/context based information using atomic contacts - the ENCoM model to study the dynamics of RNA applying it to a particular class of RNA – microRNA 125a. The authors have already shown that the model works well for proteins. The paper is well structured, the analysis is thorough and covers all important aspects of model building (dataset acquisition, benchmarking, comparison to existing methods, testing, computational cost). The performance of the model given the level of coarse-graining and computational costs, is remarkable, for the pri-Mir 125a maturation efficiency predictions. However, there are a few model/parameter choices that should be explained better and a few things that should be resolved.

Eq 2 should be explained better. What do Ni and Nj represent? What do the authors mean by interaction between atoms of types? – interaction energy? What are the values for this interaction energy for the different types?

Every atom appearing in RNA is mapped to an atom type, as such different atoms can be represented by the same atom type. There are 8 atom types and a matrix that guides the type of pairwise interaction between each combination of interacting atom types as favourable or unfavourable. As outlined in the replies to Reviewer #2, we have clarified Eq. 2. Specifically, that the interactions can only take one of two values: favorable or unfavorable. The interaction matrix has been unchanged since the work of Sobolev *et al.* (Proteins, 25(1):120–129, 1996.) and we more explicitly detailed this in the text following Eq. 2.

Nucleic acid folding and dynamics is inherently driven by different forces compared to proteins. The authors should provide more justification on why they chose to use the same parameter set for their ENCoM model that worked for proteins, on RNA as well? The protein parameters performs better than cut-ANM and PD-ANM, but wouldn't RNA optimized parameter set perform even better.

We agree that it would be fruitful to explore the question of parameter space in relation to performance on RNA, akin to what was done for proteins in the original ENCoM article. However, since the focus of the present work is more on the novel methodology to predict variant effects, we leave this

exploration for future work. In particular, we would like to explore a finer parameter space than in the original paper, with benchmarks on proteins, RNA and RNA-protein complexes, in order to determine if there is a trade-off across different types of biomolecules. We have clarified the text to explicitly mention this future goal. However, it should be noted that there is an advantage to having a single set of parameters that works, albeit perhaps not ideally, for both RNA and proteins and that is the possibility of modeling systems composed of both RNA and proteins. We have added additional details about why we think the original parameters work. Namely, the "geometric hypothesis": at such levels of coarse-graining, the most important aspect is the proper representation of global geometric features. Since ENCoM (and STeM, its ancestor) includes more detailed geometric information, it naturally performs better than ANM.

Can they demonstrate that the protein parameters when applied to RNA are for example sequence sensitive? Are G-C contacts stronger than A-U contacts. Does the model with protein parameters distinguish base-pairs, wobble pairs from mismatches?

We have added such an analysis to Figure 1. We thank you for this suggestion, and confirmed that ENCoM captures the higher rigidity of GC as opposed to AU base pairs. Moreover, Supplementary Table 7 shows that noncanonical base pairs are indeed weaker than canonical ones.

In supplementary table 1, the authors should add structures showing atom names used to assign types. e.g. Which atom in Cytosine is referred to as N5? What is the type for N1 in Uridine? Also, how do the atom types translate to the interaction strengths in the 3 bead RNA models? It will be nice to have an illustrative example – a figure - for RNA like the authors provide in [11] for proteins.

We modified Figure 1 to include the atom names. As metioned above, we have integrated a panel like the one in the original work to Figure 1.

Several RNA structures contain modified nucleotides which are structurally and functionally important. Can the authors also add stats on the numbers of structures containing modified nucleotides and the number of these altered structures that were included in their dataset and comment on the potential impact this may have on their results?

We did the analysis, 14% of the bases were replaced for the conformational change benchmark. We also clarified it affects only that specific benchmark, not the NMR ensemble variance or B-factors benchmark. We think 14% is low enough to be tolerable. Indeed, we did consider sequences with up to 10% dissimilarity as "identical" for building the pairs of conformations.

"Only the pairwise comparison between Cut-ANM and PD-ANM is statistically significant at p 0.0038 despite ENCoM having the worst average performance of the three ENMs. The reason for this is that the two flavors of ANM have a similar performance profile across the 34 sequence clusters, while ENCoM tends to perform well where they don't and vice versa." Can the authors comment on what causes this difference in performance? Are there any commonalities structural or otherwise between the cases that ENCoM yields better results?

As we mention in the text, we believe that B-factor prediction is the least relevant benchmark for our purposes. We mainly included it because it is standard in the field. In any case the individual performance is reported in the supplementary material (Supplementary Table 3). However, a more in-depth analysis of why this is observed would detract from the main goals of this work.

RMSD of 2 Angstrom seems like a very low cut off to imply that the difference is a result of a conformation change. What is the RMSD distribution for the 240 "distinct" pairs?

We agree that 2 Angstroms cutoff is on the low end. We used this to maximize the number of RNA pairs, as RNA structures are much scarcer than protein structures. This does not mean that high RMSD pairs are absent from the analysis. We have added two panels to Figure 4 to reflect this fact. The RMSD distribution ranges from 2A to 26A RMSD. Supplementary Table 4 in the text contains the detailed performance and RMSD for every pair of conformations considered.

A few minor edits: The colors are hard to see in Figs 7B and 7F. Typos in page 3, line 74, 80.

We have fixed Figure 7, it is true that the colors were hard to see without very high zoom. It should be better now. All typos were fixed.

Sincerely,

Rafael Najmanovich

Rafael Najmanovich, PhD
