# Supplementary material for: Sequence-sensitive elastic network captures dynamical features necessary for miR-125a maturation
Source: PLoS Comput Biol. 2022 Dec 14;18(12):e1010777. doi: 10.1371/journal.pcbi.1010777 (PMC9797095; doi:10.1371/journal.pcbi.1010777)
Supplement: Attachment — Submitted filename: Answer_Reviewers_R1_RJN_.pdf [file pcbi.1010777.s007.pdf]

Université de Montréal
Department of Pharmacology and Physiology
Rafael Najmanovich, PhD

Pavillon Paul-G.-Desmarais
2960 Chemin de la Tour
Montreal, H3T 1J4
QC - Canada
(514) 343-6345 or (514)-582-7552
rafael.najmanovich@umontreal.ca
https://nrglab.github.io
@RNajmanovich

November 25, 2022

Dr. Shi-Jie Chen
Academic Editor
PLOS computational Biology

Dr. Arne Elofsson Section Editor
PLOS computational Biology
Section Editor

Re: Submission of a revised version of manuscript PCOMPBIOL-D-22-00886

Dear Dr. Chen and Dr. Elofson,

We would like to thank the editors and the three reviewers for their thorough reading of our revised manuscript. As the reviewers concerns were all met, I hope that the editor will find the manuscript now suitable for publication in its present form. All figures have been run through PACE and corrected versions uploaded, all data requirements are met and we believe that the associated online documentation meets the reproducibility requirements in terms of sharing of protocols.

Reviewer 1: All previous comments have been properly addressed.
Reviewer 3: The authors have satisfactorily addressed all my comments and concerns.

Thank you.

Reviewer 2: This reviewer thanks the authors for the extensive work and for providing detailed answers to all the comments.
- Would it be possible to include the link to the code in the manuscript? (I guess it is already available at https://nrgten.readthedocs.io/)
- It would be useful for potential users to include in the documentation an example relative to the application on miRNA.
- Page 6, line 199: is purine-purine stacking less stable compared to py-py stacking?

We thank the reviewer and as per his comments above:
- We have incluided an explicit link to the code in the manuscript now.
- We have added an example relative to the application on miRNA to the documentation.
- Page 6: line 199: Purine-purine stacking is more stable than pi-pi stacking, this has been explicitly

mentioned in the text now.

Sincerely,

Rafael Najmanovich, PhD
